# Kinetic Study of OH Radical Reactions with Cyclopentenone Derivatives

**DOI:** 10.1021/acs.jpca.4c04060

**Published:** 2024-09-17

**Authors:** Patrick Rutto, Emmanuel Ubana, Talitha M. Selby, Fabien Goulay

**Affiliations:** † C. Eugene Bennett Department of Chemistry, West Virginia University, Morgantown, West Virginia 26506, United States; ‡ Department of Mathematics and Natural Sciences, University of Wisconsin-Milwaukee, West Bend, Wisconsin 53095, United States

## Abstract

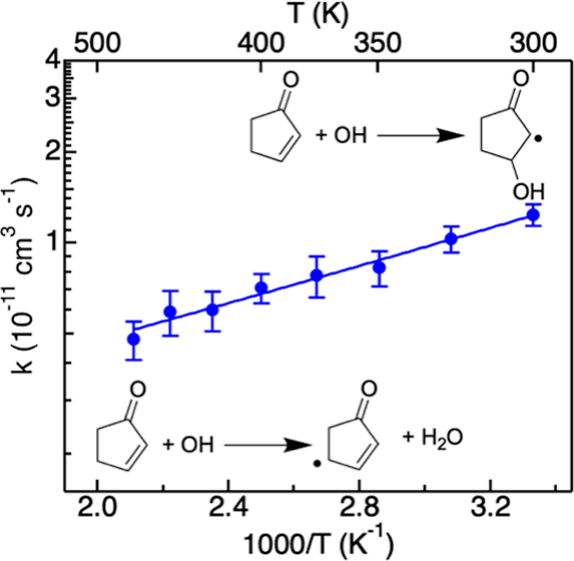

We investigated the reactions of the hydroxyl radical (OH) with cyclopentenone derivatives and cyclopentanone in a quasi–static reaction cell at 4 Torr across a 300–500 K temperature range. The OH radicals were generated using pulsed laser photolysis of hydrogen peroxide vapors, and the ketone reactants were introduced in excess. The relative concentrations of the radicals were monitored as a function of reaction time using laser-induced fluorescence. At room temperature, the reaction rate coefficients were measured to be 1.2(±0.1) × 10^–11^ cm^3^ s^–1^ for reaction with 2-cyclopenten-1-one (R1); 1.7(±0.2) × 10^–11^ cm^3^ s^–1^ for reaction with 2-methyl-2-cyclopenten-1-one (R2); and 4.4(±0. 7) × 10^–12^ cm^3^ s^–1^ for reaction with 3-methyl-2-cyclopenten-1-one (R3). Over the experimental temperature range, the rate coefficients can be fitted with the modified Arrhenius expressions *k*_1_(*T*) = 1.2 × 10^–11^ (*T*/300)^0.26^ exp (6.7 kJ mol^–1^/*R* {1/*T* – 1/300}) cm^3^ s^–1^, *k*_2_(*T*) = 1.7 × 10^–11^ (*T*/300)^6.4^ exp (27.6 kJ mol^–1^/*R* {1/*T* – 1/300}) cm^3^ s^–1^, *k*_3_(*T*) = 4.4 × 10^–12^ (*T*/300)^17.8^ exp (57.8 kJ mol^–1^/*R* {1/*T* – 1/300}) cm^3^ s^–1^. In the cases of 2-cyclopenten-1-one and 2-methyl-2-cyclopenten-1-one, the temperature dependence of the rate coefficients is similar to that calculated or measured for noncyclic conjugated ketones. We also found that the reaction with 3-methyl-2-cyclopenten-1-one was slower, with rate coefficients similar to those measured for the reaction with the saturated cyclic ketone cyclopentanone. To discuss the experimental data, we use potential energy surfaces (PES) calculated at the CCSD(T)/cc-pVTZ//M06-2*X*/6-311+G** level of theory. RRKM-based Master equation calculations were also performed to infer the most likely reaction products over a wide range of temperatures and pressures. We suggest that both abstraction and addition mechanisms contribute to the overall OH removal, forming radical products stabilized by resonance. We also discuss the relevance for combustion and atmospheric chemistry.

## 1 Introduction

Conjugated α,β-unsaturated carbonyl compounds are emitted into the atmosphere as a result of incomplete combustion of fossil fuels, biofuels,^1−6^ and industrial processes.^7−10^ They are also generated by oxidation of volatile organic compounds in the atmosphere.^11,12^ With the increasing use of ethanol-gasoline blends and biodiesel fuels as transportation energy sources,^13−15^ emissions of these potentially toxic compounds are expected to increase.^4,16,17^ Recent studies in flames have found that the oxidation of the biofuel cyclopentanone (c-C_5_H_8_O) leads to the formation of the conjugated cyclic ketone 2-cyclcopentene-1-one (C_5_H_6_O).^18,19^ Despite their importance, there is no kinetic information about the oxidation mechanisms of conjugated cyclic ketones. A full understanding of the possible advantages of using cyclic oxygenated compounds as renewable fuels requires a systematic investigation of the key elementary steps associated with their gas phase reactivity.

The hydroxyl radical (OH) acts as a potent oxidative agent in atmospheric chemistry and combustion environments.^20−24^ Its reactions with small saturated aldehydes and ketones proceed through the barrierless formation of a hydrogen-bonded intermediate.^25−27^ The transition state (TS) for the transfer of a hydrogen atom from this intermediate to the OH radical was calculated to be below the energy of the reactants for a carbonyl hydrogen from an aldehyde^28^ but well above that for an alkyl hydrogen from a ketone.^29^ The rate coefficient for the reaction of OH with acetone (2.0 × 10^–13^ cm^3^ s^–1^) at room temperature is about 2 orders of magnitude slower than that measured for reaction with acetaldehyde (1.5 × 10^–11^ cm^3^ s^–1^).^30−33^ The reaction of OH with cyclopentanone (C_5_H_8_O) is also predicted to proceed through the formation of a barrierless prereactive complex (PR).^19,25^ Although the energy barrier for successive H-abstraction is submerged for the hydrogen in α position to the carbonyl group, the measured rate coefficient remains relatively slow: 2.94 × 10^–12^ cm^3^ s^–1^ at room temperature.^34^

Reactions of OH with conjugated ketone such as pentanone,^35^ methyl vinyl ketone derivatives,^36−39^ and ethyl vinyl ketone^27^ all have room temperature rate coefficients above 1 × 10^–11^ cm^3^ s^–1^. This increase, compared to the rate for saturated ketone, suggest, that OH addition to the carbon double bond plays a significant role at room temperature. Blanco et al.^37^ correlated the overall room temperature rate coefficients for OH reactions with unsaturated ketones to the energy of the highest occupied orbitals. This reactivity trend suggests that methyl substitution on the carbon double bond increases the reactivity of the unsaturated ketone. Computational studies show that although OH-addition is the dominant mechanism at room temperature, abstraction is not negligible.^26,27^ Barriers to the formation of addition products are submerged, while they range from low to submerged for the formation of the abstraction products.

The back dissociation of the OH–conjugated molecule prereactive association complex as the temperature increases led to a decrease of the addition rate coefficient near and above room temperature, while that for abstraction increased. The effective reaction rate coefficient reached a minimum value between 500 and 1000 K.^40−44^ The overall temperature dependence of the rate coefficient over the 300–2000 K temperature range depends on conjugation, stereoisomerism, and alkyl-group substitution.^42^ The temperature at which the effective rate coefficient is minimum was found to be much lower for OH reactions with conjugated ketones^43,44^ than for reactions with conjugated hydrocarbons.^42^ This difference is due to a lower OH-addition rate coefficient near room temperature for conjugated carbonyl compounds compared with that for conjugated hydrocarbons.^42,44^ Although estimated values are available for the reactions of OH with a series of conjugated ketone molecules,^45^ researchers have not yet systematically investigated the effects of substitution on the temperature dependence of the OH reaction rate coefficient with conjugated ketones.

Through abstraction or addition, the initial attack of the OH radical on conjugated ketones results in the formation of a carbon-centered radical intermediate. If the resulting unpaired electron is in α–position, relative to the carbonyl group, the radical product becomes a substituted vinoxy radical. The vinoxy radical and its derivatives are known to play a role in combustion and atmospheric processes.^46−49^ The methylvinoxy radical (or acetonyl radical) is another important intermediate in atmospheric and combustion chemistry processes.^50,51^ These radicals have distinctive properties and reactivity due to the potential delocalization of the radical over the carbonyl group.^52−58^ In the case of the ground state vinoxy radical, however, the resonance structure with the radical on the carbon atom is expected to be the dominant one,^48^ leading to a lower resonance stabilization compared to noncarbonyl resonance-stabilized hydrocarbon radicals.^53^ Nonetheless, vinoxy-type radicals are predicted to have a lower reactivity toward molecular oxygen.^47^ Kinetic measurements of the reaction of vinoxy and the methyl-substituted vinoxy radical with O_2_ show high-pressure limit rate coefficient^59^ below 1 × 10^–12^ cm^3^ s^–1^ while they are close to 1 × 10^–11^ cm^3^ s^–1^ for the vinyl radical.^60^ This is likely due to the back dissociation of the peroxy-radical.^47,61^ A full understanding of the reactivity of conjugated carbonyl compounds requires a systematic investigation of the effect of vinoxy-like intermediate formation on the oxidation mechanisms.

Table 1 shows the structure of 2-cyclopentene-1-one (cyclopentenone) and its methyl derivatives 2-methyl-2-cyclopenten-1-one (2-methyl-cyclopentenone) and 3-methyl-2-cyclopenten-1-one (3-methyl-cyclopentenone), along with the saturated cyclopentanone. The rate coefficient for OH reactions with cyclopentanone (R4) has been measured at room temperature^34^ and high temperatures and has been found to decay monotonically over the 300–1000 K temperature range.^62^ However, kinetic data are not available for the reaction of cyclic conjugated ketones with the OH radical (R1: OH + cyclopentenone, R2: OH + 2-methyl-cyclopentenone, R3: OH + 3-methyl-cyclopentenone).
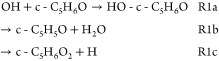

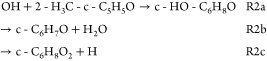

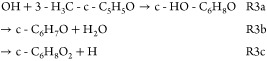

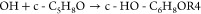


**Table 1:**
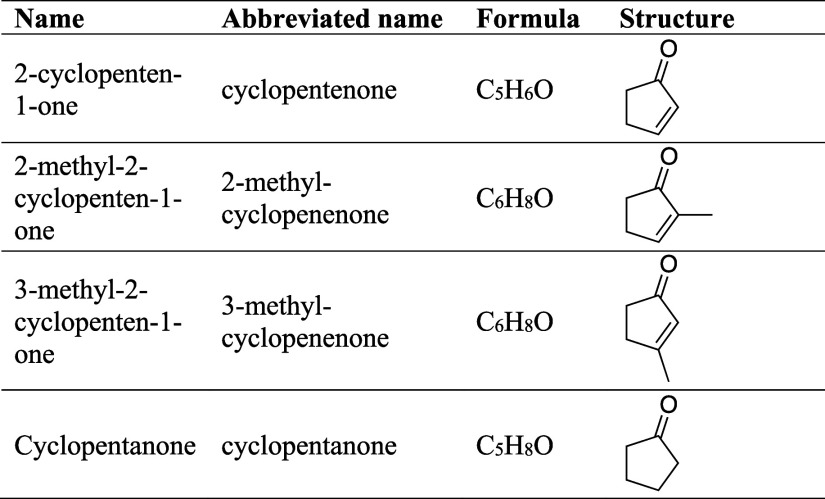
Cyclopentenone Derivatives and Cyclopentanone

The present study aims to understand the role of conjugation and methyl substitution during the reaction of the OH radical with the conjugated cyclic ketones displayed in Table 1. Rate coefficients for reactions R1, R2, and R3 were measured over the 1–10 Torr pressure range and the 300–500 K temperature range using a pulsed laser photolysis and laser-induced fluorescence setup. For comparison, we investigated the reaction of the OH radical with the saturated cyclopentanone over the same temperature range. Potential energy surfaces of reactions R1 to R3 (channels a and c) were calculated at the CCSD(T)/cc-pVTZ//M06-2X/6-311+G** level of theory to further understand the effect of vinoxy-like radical formation on the reaction mechanisms and kinetics. RRKM-based Master equation calculations were also performed to predict the most likely addition products formed by the reactions. TSs for abstraction channels (c) were calculated at the same level of theory. We found that both the conjugation and the methyl substitution have a significant effect on the measured values of the rate coefficient and on their dependence on temperature. Over a broad range of temperatures and pressures, we expected the reaction products to be resonance-stabilized radicals and substituted vinoxy radicals.

## 2 Experimental Procedure

The kinetic measurements were conducted in a pressure- and temperature-regulated stainless steel reaction cell over a pressure range of 1–10 Torr and a temperature range of 300–500 K. Only an abbreviated description is presented here, as the experimental apparatus has been described in detail elsewhere.^63−65^

The system has five optical access ports: four horizontal ports for laser access, and one vertical port for the photomultiplier tube (PMT). The four laser windows have uncoated UV fused silica at Brewster angle (53°) to reduce laser beam reflections. The OH radical was produced by 266 nm photolysis using the fourth harmonic of a Nd:YAG laser. It was detected as a function of time by exciting the A^2^Σ–X^2^Π (1,0) vibronic band at 282 nm using a frequency-doubled dye laser and by collecting the A^2^Σ–X^2^Π (1,1) fluorescence using a PMT equipped with a 305 nm band-pass filter. The time-resolved fluorescence was integrated over a 1 μs time window 500 ns after the probe laser using a Boxcar integrator (SRS250). The temporal profiles of the OH radicals were obtained by averaging at least 10 laser shots and changing the time delay between the pump and probe lasers using a delay generator (SRS DG535) from −50 to 1500 μs with 10 μs steps, relative to the photolysis laser.

We introduced the conjugated ketones in the flow cell by bubbling a regulated amount of argon buffer through a liquid sample. The temperature of the sample was maintained at 17 °C using a chiller to avoid any condensation in the room temperature lines. The vapor pressures for all the reactants were measured in the laboratory at 17 °C (Figure S1). The reactant number densities are determined directly from the known vapor pressures, gas flow rates, and the measured bubbler total pressure. The uncertainties on the calculated number densities are estimated to be of the order to 10%, mostly due to the uncertainties on the vapor pressure and bubbler pressure measurements. The cyclic ketones used in these experiments were purchased from Sigma-Aldrich and had stated purities of 98%, and the argon buffer gas was from Matheson Tri-Gas, labeled Ultra High Purity grade, 99.999%.

The gaseous reactants and the bath gas were introduced into the flow using calibrated mass flow controllers (MKS instruments). Before entering the reaction cell, the gases were premixed and preheated in a 50 cm^3^ mixture chamber. We heated the reaction cell using a heating mantle connected to controllers and solid-state relays for regulation. The temperature in the reaction cell was controlled using a type-K thermocouple inserted near the laser overlap region and validated by CN LIF.^64^ Pressures in the reaction cell were regulated from 1 to 10 Torr using a manual butterfly valve and monitored using 10 Torr capacitance manometers (Baratron, MKS instruments). The gas flow was kept sufficiently high to refresh the gas mixture every laser pulses.^66^

## 3 Theoretical Calculations

### 3.1 Potential Energy Surfaces

We performed Electronic Structure calculations using the Gaussian 16 series of programs.^67^ Optimized geometries of all stationary points on the potential energy surface were obtained by density functional theory (DFT) with the hybrid exchange–correlation functional M06-2X^68^ and the 6-311+G** basis set with ultrafine grid and ultratight convergence criteria.^69,70^ First-order saddle points were found by scanning bond lengths or angles between intermediates. TSs were confirmed by the presence of an imaginary frequency. We also performed Intrinsic Reaction coordinate calculations at the M062*X*/6-311+G** level of theory to confirm the validity of the TSs. Species on the PES were ensured to be the minimum energy conformers by 1-D relaxed scans of dihedrals at the same level of theory. Final single-point energy calculations were performed using the CCSD(T)/cc-pVTZ level of theory and corrected for zero-point energy from the M062*X*/6-311+G** calculations. We used T1 diagnostic values to assess the multireference character of all stationary points (see Tables S1 and S2). Values were less than 0.02 for closed-shell species and less than 0.045 for open-shell species, indicating that multireference effects may be ignored for all electronic structures.^71,72^ The atomic coordinates of the optimized structures, rotational constants, and vibrational frequencies for all stationary points are reported in Tables S3–S5.

### 3.2 Master Equation Calculations

We calculated product branching fractions for the OH-association entrance channels over the 300–1000 K and 1–1000 Torr temperature and pressure ranges using the open-source Master equation solver for multi-energy well reactions (MESMER). Given stationary points of the potential energy surface, MESMER solves coupled differential equations describing reaction and energy transfer kinetics. This methodology was previously described in detail by Glowacki et al.^73^ and has been employed to investigate radical reactions with unsaturated hydrocarbons^74,75^ and NH_3_.^76^

The classical rotor approximation was used for the reactants, whereas quantum rotors were used for the products. The energy grain size for cyclopentenone and 2-methylcyclopentenone was 50 cm^–1^,^74,75^ while an increased energy grain size of 100 cm^–1^ was used for 3-methyl-cyclopentenone, partly due to the complexity of the potential energy surface. The energy grain was set to span a region possessing 20 *k*_B_*T* of energy above the highest stationary point energy, where *k*_B_ is the Boltzmann constant and *T* is the temperature. The energy transfer and Lennard-Jones parameters (ε and σ) for the intermediates were 4.3 Å and 380 cm^–1^. These values were taken from previous work on OH reactions with unsaturated hydrocarbons.^74^ For the bath gas, the energy transfer parameter (ε) was 2.55 Å and the Lennard-Jones parameter was 10.2 cm^–1^.^77−79^ The collisional energy transfer parameter Δ*E*_down_ was kept independent of temperature at Δ*E*_down_ = 250 cm^–1^. Calculations were also performed at Δ*E*_down_ = 300 cm^–1^, as suggested by Shannon et al.^77^ Lower values of Δ*E*_down_ have no significant effect on the calculation outcomes.^74,75^ After performing the calculations, we observed that the branching fractions were independent of the energy grain size and energy transfer parameters within the 300–1000 K and 4–1000 Torr temperature and pressure ranges.

## 4 Experimental Results

### 4.1 Pseudo-First-Order Kinetics

The rate coefficients (*k*_1_, *k*_2_, *k*_3_, and *k*_4_) for reactions R_1_, R_2_, R_3_, and R4 were determined through pseudo-first-order kinetics with the cyclic ketone reactant being in excess compared to the laser-generated OH radical. The temporal profile of the OH radical relative concentration follows eqs 1 and 2
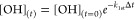
1
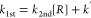
2where [*R*] is the reactant’s number density, *k*_1st_ is the pseudo-first-order rate coefficient, and *k*_2nd_ is the second-order rate coefficient for the reaction between the radical and the excess reactant. [OH]_(*t*=0)_ and [OH]_(*t*)_ are the OH radical number densities initially in the reaction zone and at a delay time Δ*t*. The first-order rate coefficient *k*′ accounts for reactions of OH with hydrogen peroxide and impurities.

**Figure 1:**
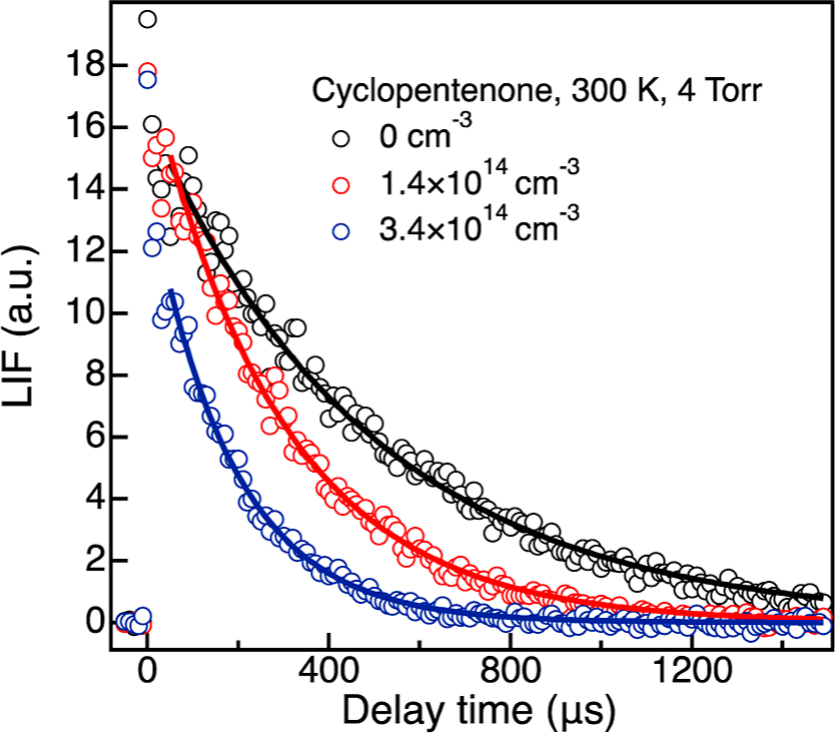
OH LIF decay profiles measured at 300 K and 4 Torr for different cyclopentenone number densities. The lines are exponential fits to the experimental data.

Figure 1 shows LIF decay profiles measured at 300 K for OH + cyclopentenone for given reactant number densities. The decays are representative of the recorded LIF profiles for all reactants across the entire pressure and temperature ranges. Given a specific reactant number density, *k*_1st_ was determined by fitting the integrated LIF signal against the delay time using eq 1 (lines in Figure 1) from 50 to 1500 μs after the pump laser pulse. Earlier times were not included in the fit to avoid any effect from the collisional relaxation of the radicals.

**Figure 2:**
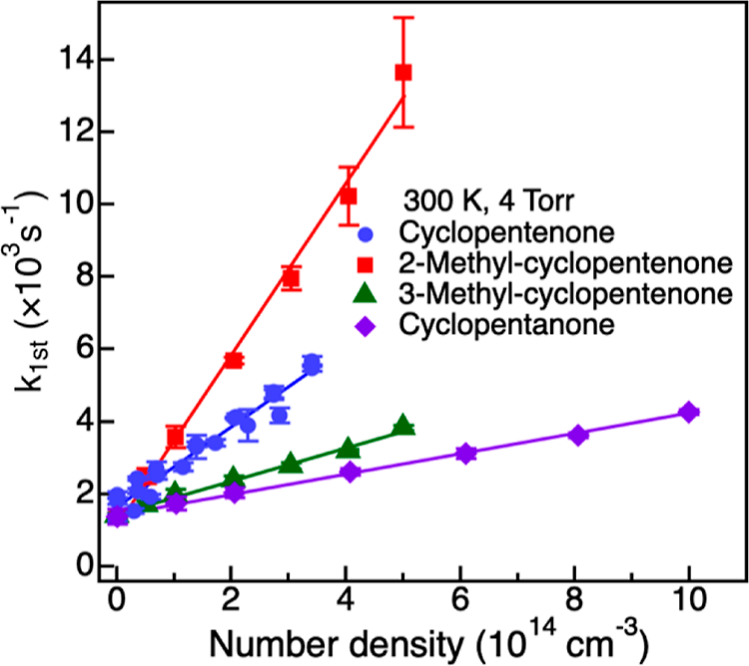
Second-order plot for the reaction of the OH radical with cyclopentenone, 2-methyl-cyclopentenone, 3-methyl-cyclopentenone, and cyclopentanone, recorded at 300 K and 4.0 Torr. The data are reported with 2-standard deviation uncertainty from the average of at least 3 independent data sets. The solid lines are linear fit to the data.

Figure 2 displays the *k*_1st_ values as functions of reactant number densities for all reactants, recorded at 300 K and 4.0 Torr. Each data point represents the mean of a minimum of three separate data sets with 2σ error bars. The overall second-order rate constant was derived by fitting the data with eq 2. The good linearity of the data points confirms the validity of the pseudo-first-order approximation. The measured rate coefficients were not found to be dependent on pressures within the 1–10 Torr range at room temperature (Figure S2).

### 4.2 Temperature Dependence of the Reaction Rate Coefficients

**Figure 3:**
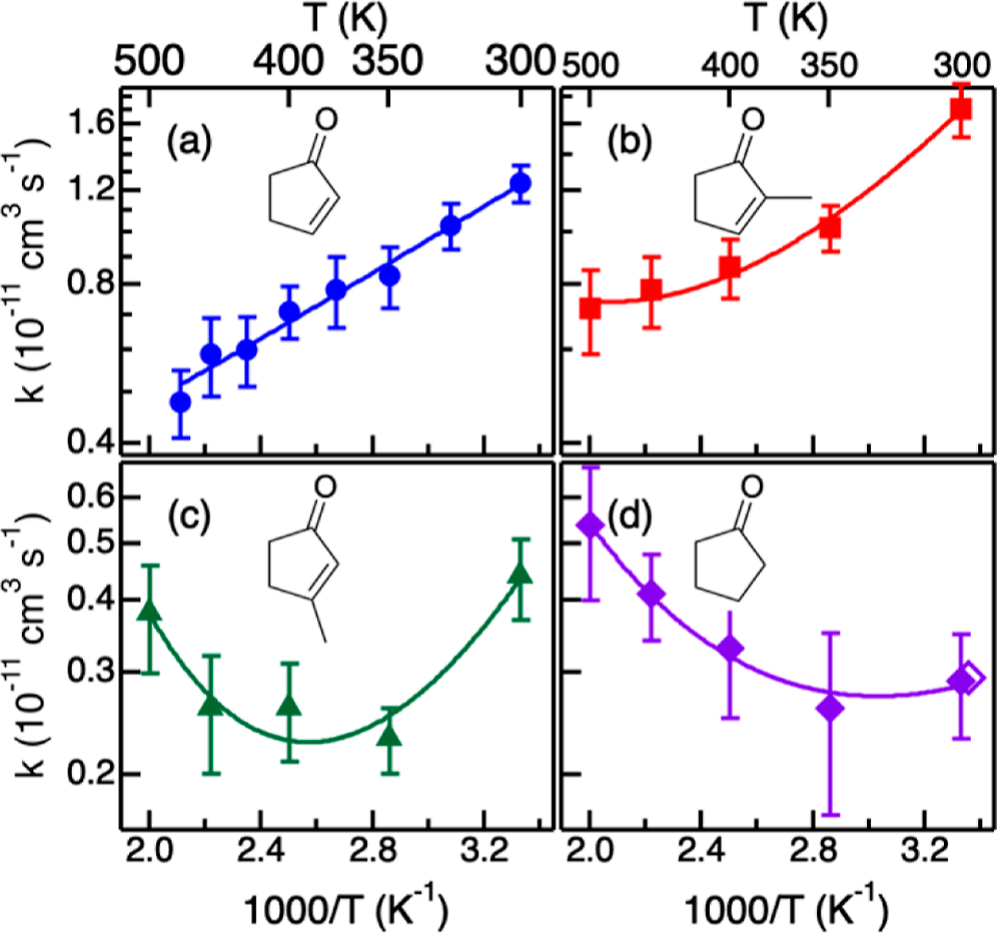
Arrhenius plots for reactions of the OH radical with (a) cyclopentenone, (b) 2-methyl-cyclopentenone, (c) 3-methyl-cyclopentenone, and (d) cyclopentanone at 4 Torr. The error bars are the sum of the statistical uncertainties (2 standard deviations from the average of at least 3 independent data sets) and experimental uncertainties (10%). Solid lines are fitted lines obtained using a modified Arrhenius equation within the 300–500 K temperature range.

Figure 3 displays Arrhenius plots for the reactions of OH with (a) cyclopentenone, (b) 2-methyl-cycploentenone, (c) 3-methyl-cyclopentenone, and (d) cyclopentanone, recorded from 300 to 500 K at 4 Torr. Each data point is the average of at least three independent experiments at a given temperature. The error bars are the sum of the statistical uncertainties (2 standard deviations from the average of at least 3 independent data sets) and experimental uncertainties (10%). The experimental data in Figure 3 are fit with a modified Arrhenius equation
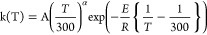
3where *k*(*T*) is the rate coefficient at temperature *T*, *R* is the gas constant, and *A*, α, and *E* are fit parameters. The rate coefficient values, number density ranges, and fit parameters are displayed in Table 2. The fit parameters displayed in Table 2 are representative of the measured reaction rate coefficients over the investigated experimental range and should not be used to predict values at higher or lower temperatures.

**Table 2:** Experimental Conditions, Rate Coefficients, and Fit Coefficients for Reactions of OH with Cyclopentenone Derivatives and Cyclopentanone^a^

				fit coefficients		
	temperature (K)	number density (10^13^ cm^–3^)	*k* (10^–11^ cm^3^ s^–1^)	*A* (10^–11^ cm^3^ s^–1^)	α	*E* (kJ/mol)
cyclopentenone	300	3.5–34	1.2 ± 0.1	1.2	0.26	–6.7
	325	2.9–33	1.0 ± 0.1			
	350	2.9–28	0.83 ± 0.11			
	375	2.6–26	0.78 ± 0.12			
	400	2.5–24	0.71 ± 0.08			
	425	2.4–23	0.60 ± 0.09			
	450	1.9–18	0.59 ± 0.10			
	475	1.8–17	0.48 ± 0.07			
2-methyl-cyclopentenone	300	7.0–69	1.7 ± 0.2	1.7	6.4	–25.6
	350	5.8–56	1.0 ± 0.1			
	400	4.7–46	0.86 ± 0.11			
	450	3.9–38	0.78 ± 0.12			
	500	3.4–34	0.72 ± 0.13			
3-methyl-cyclopentenone	300	5.1–50	0.44 ± 0.07	0.44	17.8	–57.5
	350	4.4–43	0.23 ± 0.03			
	400	3.5–34	0.26 ± 0.05			
	450	3.0–31	0.26 ± 0.06			
	500	2.4–24	0.38 ± 0.08			
cyclopentanone	300	1.0–100	0.29 ± 0.06	0.29	9.10	–25.0
	350	8.1–79	0.26 ± 0.09			
	400	6.7–67	0.33 ± 0.08			
	450	6.5–63	0.41 ± 0.07			
	500	6.5–63	0.54 ± 0.14			

a Uncertainties are the sum of the statistical uncertainties (2 standard deviations from the average of at least 3 independent datasets) and experimental uncertainties (10%).

The room temperature rate coefficient for OH reacting with cyclopentanone, 2.9(±0.6) × 10^–12^ cm^3^ s^–1^, is in excellent agreement with previous measurements: 2.94(±0.02) × 10^–12^ cm^3^ s^–1^ (open diamond in Figure 3d).^34^ As the temperature increases the measured values also increases, reaching 5.4(±0.1) × 10^–11^ cm^3^ s^–1^ at 500 K. At room temperature, the rate coefficients for reactions of OH with cyclopentenone, 1.2-(±0.1) × 10^–11^ cm^3^ s^–1^, and 2-methyl-cyclopentenone, 1.7 (±0.2) × 10^–11^ cm^3^ s^–1^, are both at least four times greater than that for the saturated cyclopentanone. In both cases, the rate was found to decrease with increasing temperature over the whole experimental temperature range. The reaction rate coefficient for the reaction of OH with 3-methyl-cyclopentenone is systematically lower than that of the other 2 conjugated ketones. Within the experimental error bars, it decreases first as the temperature increases to reach a minimum value of 0.23(±0.03) × 10^–11^ cm^3^ s^–1^ at 350 K. At 500 K, the value increased to 0.38(±0.08) × 10^–11^ cm^3^ s^–1^.

## 5 Potential Energy Surfaces

**Figure 4:**
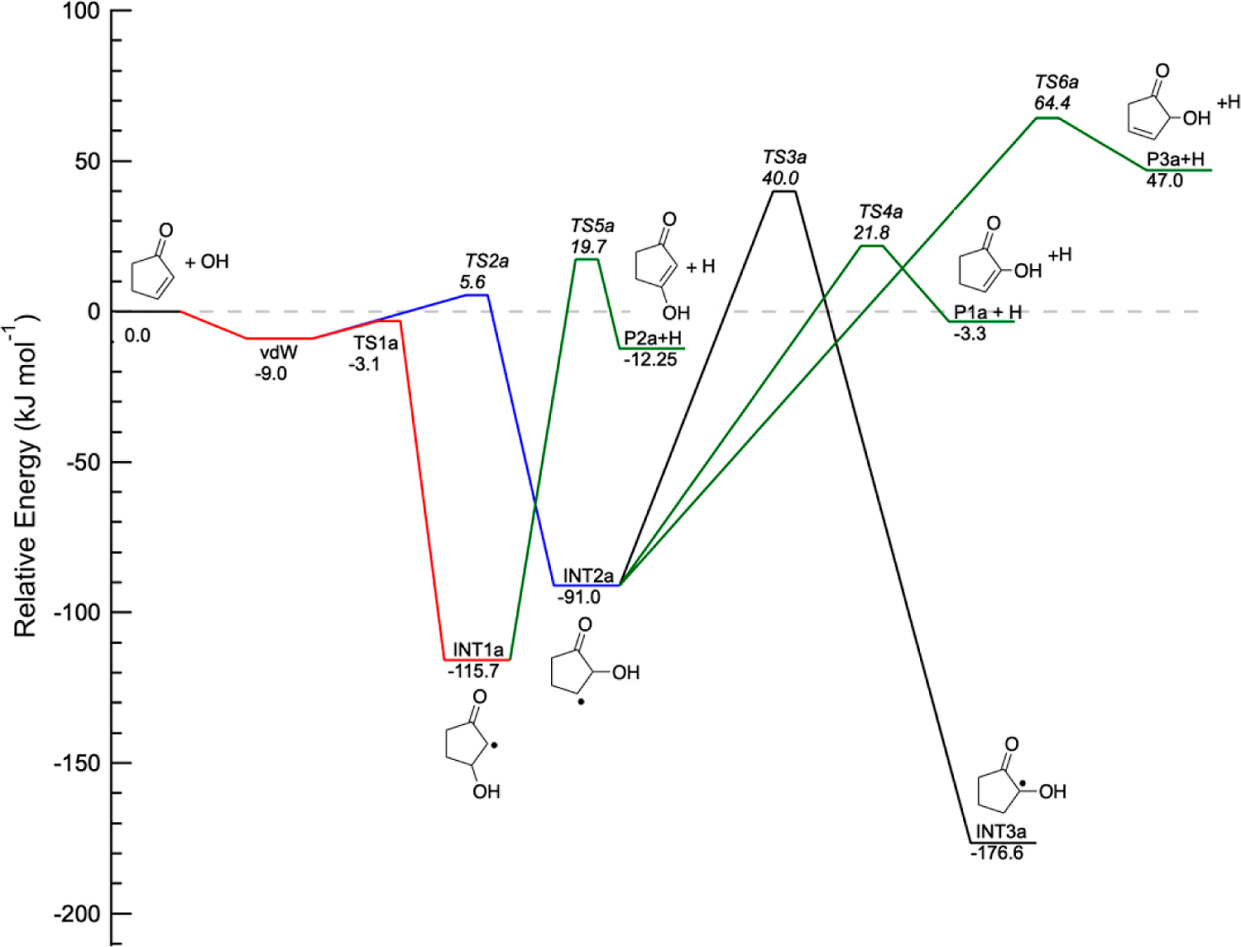
Part of the potential energy surface for the addition channels of the cyclopentenone + OH reaction calculated using the CCSD(T)/cc-pVTZ//M06-2*X*/6-311+G**level of theory. The energies, corrected for zero-point energy (0 K), are given in kJ mol^–1^.

To support the interpretation of the kinetic data, parts of the potential energy surfaces (PES) for reactions of OH with substituted cyclic conjugated ketones have been computed using the CCSD(T)/cc-pvTZ//M06-2*X*/6-311+G** level of theory. Figure 4 displays the PES for OH addition onto cyclopentenone. The reaction proceeds through the initial barrierless formation of a PR through van der Waals interactions, 9.0 kJ mol^–1^ below the energy of the reactants. The adduct can isomerize to two addition intermediates via TS1a and TS2a, at −3.1 kJ mol^–1^ and 5.6 kJ mol^–1^, respectively, relative to the reactants. Addition of the OH onto carbon 3 of cyclopentenone to form INT1a is energetically more favorable than addition onto carbon 2 by 24.7 kJ mol^–1^. The increased stability of INT1a is likely due to the formation of a vinoxy-like radical.

The delocalization of the radical is also likely to decrease the energy of TS1a relative to TS2a. Several isomerization and dissociation pathways are available from INT1a and INT2a through TSs above the energy of the reactants. INT2a can isomerize through H-transfer to an OH-substituted vinoxy radical, INT3a, located 176.6 kJ mol^–1^ below the energy of the reactants. Although INT3a is the most stable intermediate on the calculated PES, the TS leading to its formation from INT2a is 40.0 kJ mol^–1^ above the energy of the reactants. Elimination of an H atom to form OH-substituted cyclopentenone isomers is found to be slightly exothermic for conjugated products 2-hydroxycyclopent-2-ene-1-one (P1a) and 3-hydroxycyclopent-2-ene-1-one (P2a), while it is endothermic for the nonconjugated product 2-hydroxycyclopent-3-ene-1-one (P3a).

**Figure 5:**
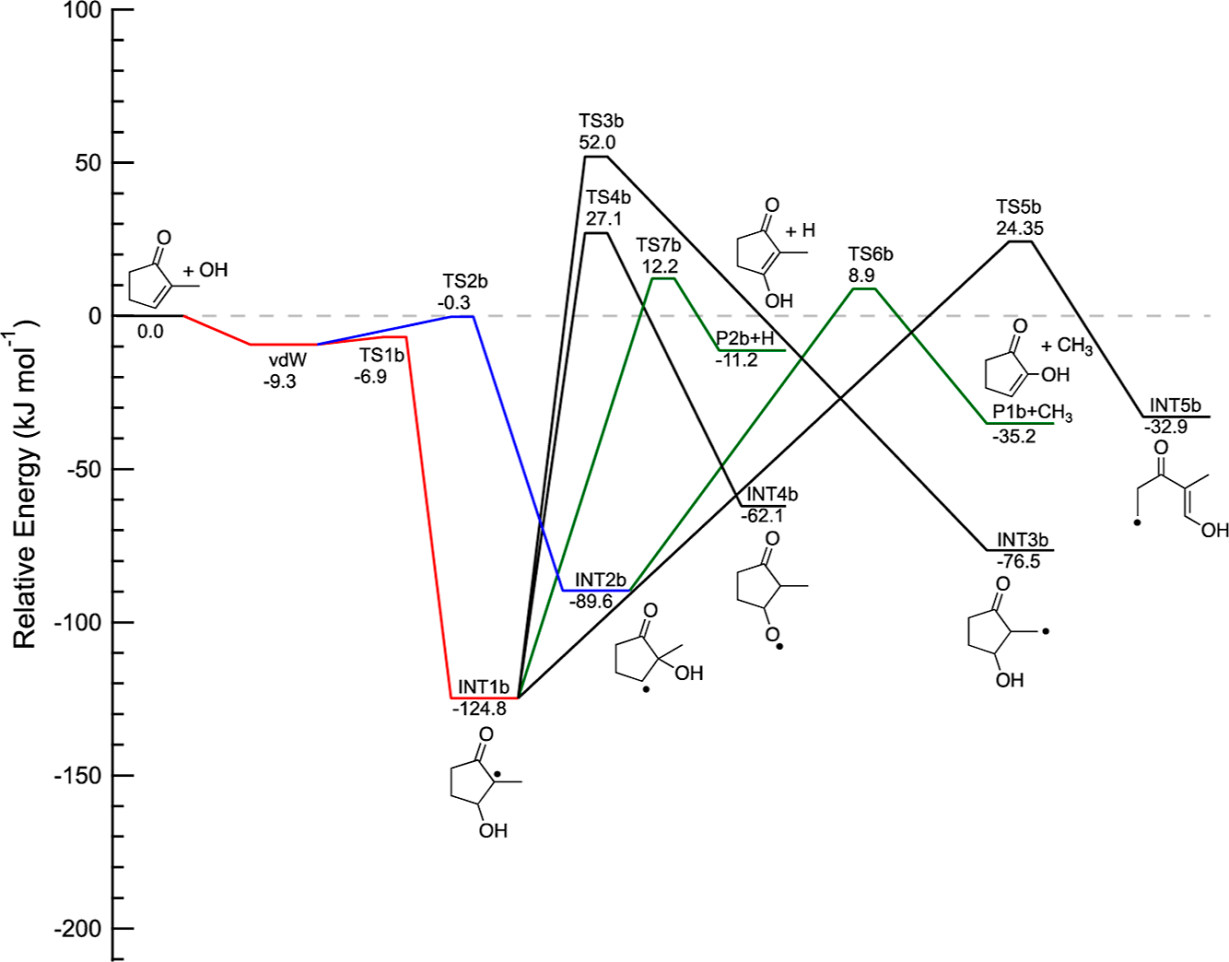
Part of the potential energy surface for the addition channels of the 2-methyl- cyclopentenone + OH reaction calculated using the CCSD(T)/cc-pVTZ//M06-2*X*/6-311+G**level of theory. The energies, corrected for zero-point energy (0 K), are given in kJ mol^–1^.

Figure 5 displays the PES for OH addition onto 2-methyl-cyclopentenone. The TSs TS1b and TS2b for the formation of the two substitution intermediates (INT1b and INT2b) are both below the energy of the reactants. INT1b, formed by addition onto carbon 3, is lower in energy than the corresponding intermediate in Figure 4, likely due to stabilization of the vinoxy radical by the electron-donating methyl group and hyperconjugation stabilization.^80^ We found that the methyl substitution has no significant effect on the energy of INT2a. The TSs for isomerization or H- and CH_3_-elimination are above the energy of the reactants. Methyl elimination from INT2b has the smallest energy barrier, leading to P1b + CH_3_, 35.2 kJ mol^–1^ below the energy of the reactants. H-elimination from INT1a is also exothermic by 11.2 kJ mol^–1^. H-transfer and ring opening pathways are all exothermic with TSs at least 24 kJ mol^–1^ above the energy of the reactants.

**Figure 6:**
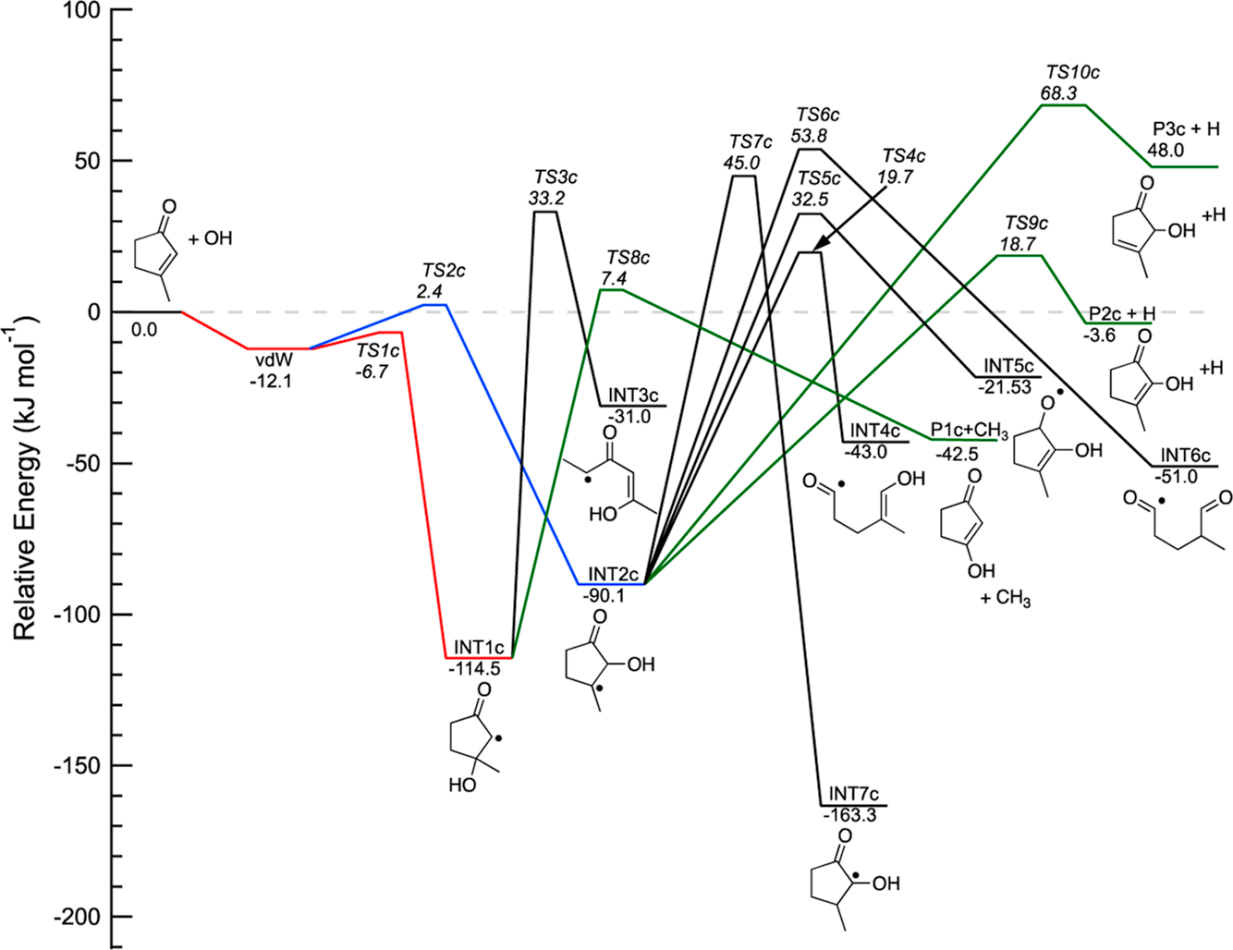
Part of the potential energy surface for the addition channels of the 3-methyl-cyclopentenone + OH reaction calculated using the CCSD(T)/cc-pVTZ//M06-2*X*/6-311+G**level of theory. The energies, corrected for zero-point energy (0 K), are given in kJ mol^–1^.

Figure 6 displays the PES for OH addition onto 3-methyl-cyclopentenone. Although the initial van der Waals adduct is slightly more stable than that for the reaction with cyclopentenone and 2-methyl-cyclopentenone, the addition intermediates have more similar energies than in the case of cyclopentenone. All TSs for isomerization or H- and CH_3_-elimination from INT1c and INT2c are above the energy of the reactants. The TS for isomerization of the vdW adducat to INT2c is calculated to be only 2.4 kJ mol^–1^ above the energy of the reactants. The H- and CH_3_-elimination pathways are all exothermic, exepct for P3c, with methyl elimination having the lowest energy barrier of all the bimolecular channels. The OH-substituted vinoxy radical INT7c is the lowest energy intermediate, 163.3 kJ mol^–1^ below the energy of the reactants.

## 6 Discussion

**Figure 7:**
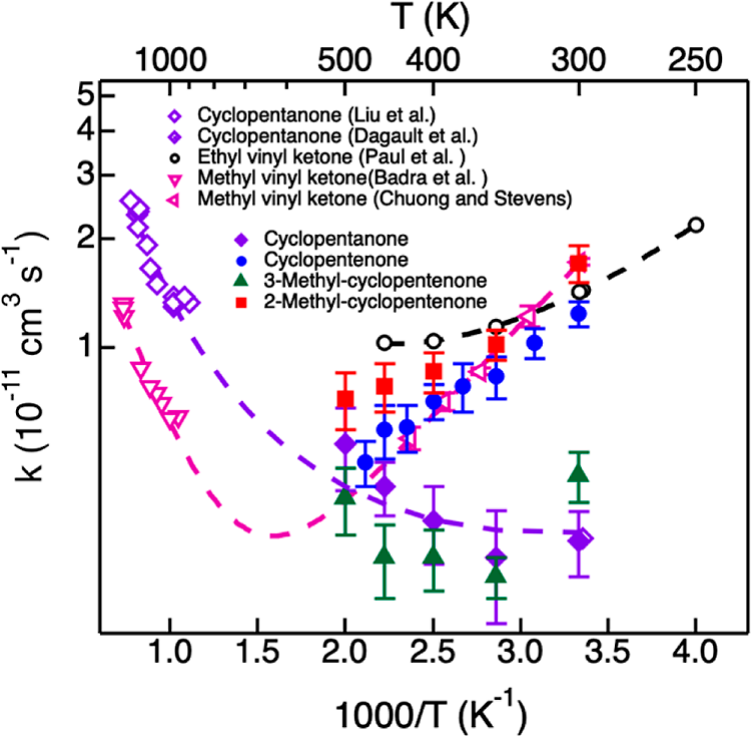
Arrhenius plots for the reaction of OH with cyclopentanone (purple diamonds), cyclopentenone (blue dots), 2-methyl-cyclopentenone (red squares), 2-methyl-cyclopentenone (green triangles), methyl vinyl ketone (inverted pink triangles), and ethyl vinyl ketone (black dots). Open markers are data from the literature.^27,34,43,44,62^ Lines are modified Arrhenius fits to the data.

Figure 7 is an Arrhenius plot for OH reactions with cyclopentanone, cyclopentenone, and the two methylated cyclopentenones. Available experimental data for the reactions of OH with methyl vinyl ketone^43,44^ and cyclopentanone^34,62^ are also displayed, along with theoretical data for the reaction with ethyl vinyl ketone.^27^ The dashed lines are fit to the literature data using a modified Arrhenius equation. At room temperature, OH reactions with cyclopentenone and 2-methyl-cyclopentenone are 4 times faster than the reaction with cyclopentanone. The rate coefficients decrease with increasing temperature to reach values close to that of OH + cyclopentanone at 500 K. This trend is similar to previous measurements and calculations on OH reactions with methyl vinyl ketone^44^ and ethyl vinyl ketone.^27^

The kinetic data in Figure 7 suggest that near room temperature, OH reactions with cyclopentenone and 2-methyl-cyclopentenone are governed by the initial OH association with the C=C double bond, followed by isomerization to addition intermediates. The submerged addition barriers for the formation of INT1(a and b), as displayed in Figures 4 and 5, are consistent with the temperature dependence observed for cyclopentenone and 2-methyl-cyclopentenone. Paul et al.^27^ calculated that at room temperature, addition accounts for 77% of the overall OH + ethyl vinyl ketone mechanism. Assuming a similar mechanism for cyclopentenone and 2-methyl-cyclopentenone, it is expected that association/addition is also the dominant mechanism for these cyclic conjugated ketones at and near room temperature. The formation of a methyl-substituted vinoxy radical may contribute to the slightly faster reaction rate measured in the case of 2-methyl-cyclopentenone compared to cyclopentenone. As the temperature increases, the association adduct is likely to dissociate back to the reactants. As predicted for ethyl vinyl ketone, abstraction products are likely to represent more than half of the total reaction products at 500 K.^27^

The lower rate coefficient (<1 × 10^–11^ cm^3^ s^–1^) measured for the reaction of OH with 3-methyl-cyclopentenone, compared to that measured for 2-methyl-cyclopentenone, may suggest a slower association mechanism as observed for other conjugated carbonyl molecules.^81^ The potential energy surface displayed in Figure 6 shows a small energy barrier for the formation of INT2c. At room temperature, isomerization of the vdW adduct to INT2c is therefore likely to be slower than isomerization to INT1c through a submerged barrier. A similar trend is expected for OH reactions with cyclopentenone. In the case of 2-methyl-cyclopentenone, the barrier for INT2b formation is lower, making this pathway more likely. At room temperature, the association of the OH radical onto cyclopentenone and 3-methyl-cyclopentenone must proceed mostly through isomerization into INT1a and INT1c, respectively. The presence of a methyl group on the carbon in β-position of the carbonyl group may create a steric hindrance that could make the formation of the vinyl-like radical INT1c less favorable.^42^ The combination of these enthalpic (INT2c) and entropic (INT1c) hindrances may explain the low effective rate coefficient measured for OH reactions with 3-methyl-cyclopentenone. Further investigations into the effects of methyl substitutions in β-position are required to fully explain the observed trends.

**Figure 8:**
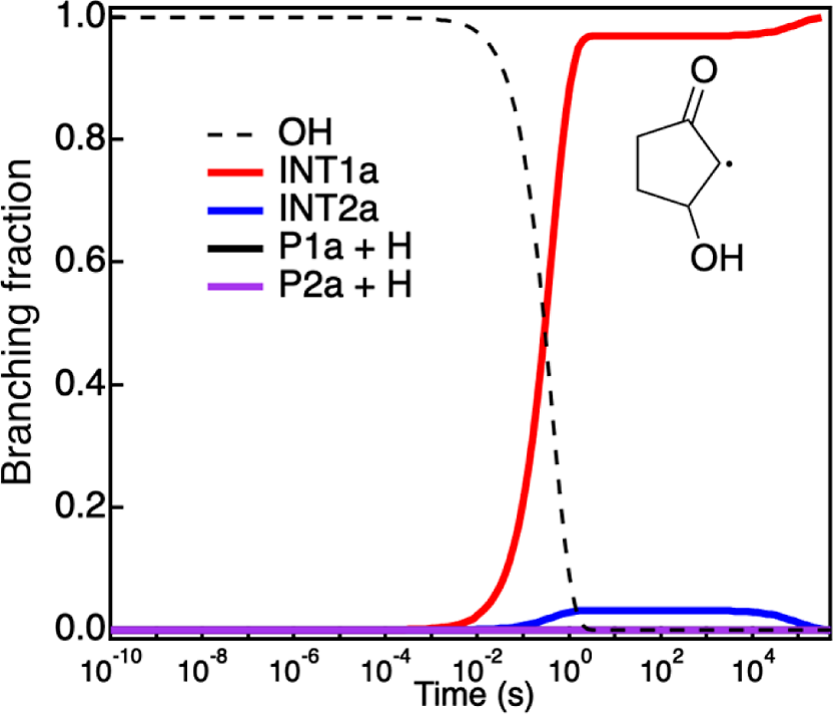
MESMER-calculated temporal profiles of INT1a (red line), INT2a (blue line), P1a (black line), and P2a (purple line) from reactions of the OH radical with cyclopentenone at 298 K and 5 Torr.

Product distributions for OH reactions with cyclopentenone and 2-methyl-cyclopentenone will be dominated by addition products near room temperature. Their product branching ratios have been predicted by solving the RRKM-based Master equation. However, at higher temperatures, abstraction products will dominate, and addition products are likely to become negligible. In the case of 3-methyl-cyclopentenone, because abstraction is suggested as the dominant channel, addition products are likely to be negligible even at room temperature and below. Figure 8 displays the calculated branching ratios at 298 K for the formation of INT1a (red line), INT2a (blue line), P1 + H (black line), and P2 + H (purple line) from the initial association of the OH radical onto cyclopentenone. The other intermediates and products are predicted to be formed in negligible amounts. At room temperature, the main reaction products are the two addition intermediates, INT1a and INT2a. Once formed through submerged barriers, the intermediates are stabilized by collision with the surrounding gas. At 500 K (Figure S2), the stabilized addition intermediates are also the dominant products, although, over a longer reaction time, slow isomerization pathways lead to the formation of P1a and P2a through H-elimination. These channels are however expected to be negligible as abstraction is the dominant mechanism at these temperatures. Pressure is found to have no effect on the predicted branching ratios from 1 to 1000 Torr. Calculated product branching fractions for 2-methyl-cyclopentenone and 3-methyl-cyclopentenone show similar trends (Figures S3 and S4), with the loss of the methyl group being the dominant exit channel at higher temperatures.

As observed for the reaction with methyl vinyl ketone,^44^ rate coefficients for OH reactions with cyclopentenone and 2-methyl-cyclopentenone are likely to reach a minimum value between 600 and 800 K and to increase at higher temperatures. The high temperature trend is characteristic of an abstraction mechanism with negligible contribution from the addition channels. Table 3 displays the energy of the products, TSs, and the pre- and postreactive complexes (PO) for the H-abstraction channels of cyclopentanone,^25^ cyclopentenone, 2-methyl-cyclopentenone, and 3-methyl-cyclopentenone. For the cyclic conjugated ketones, the most thermodynamically favorable abstraction products resulted from the abstraction of a hydrogen atom on carbon 4, in β-position of the carbonyl. The formed radical has both allyl-like and vinoxy-like character leading to a super-resonance stabilization. The trend is inverted for abstraction on cyclopentanone due to the lack of resonance stabilization following abstraction in β-position. Abstraction of an H atom from a methyl group also led to stabilization due to the allyl-like character of the radical products.

**Table 3:** CCSD(T)/cc-pVTZ//M06-2*X*/6-311+G** Energies of the PR, TS, PO and Products for abstraction Channels of the OH Reactions with Cyclopentenone Derivatives

reactant	position	PR complex (kJ mol^–1^)	TS (kJ mol^–1^)	PO complex (kJ mol^–1^)	products (kJ mol^–1^)
cyclopentanone^25^	C4	–28.1	0.6	–98.0	–89.6
	C5	–28.1	4.10	–137.3	–116.7
cyclopentenone	C4	–3.75	11.0	–143.8	–130.3
	C5	–11.0	8.5	–120.2	–92.0
2-methyl-cyclopentenone	C4	–6.6	9.3	–142.2	–131.7
	C5	–31.9	7.9		–93.0
	methyl	–31.0	2.5	–129.5	–104.0
3-methyl-cyclopentenone	C4	–9.3	7.7	–136.3	–123.4
	C5	–26.1	7.3	–119.0	–90.4
	methyl	–13.5	12.9	–126.7	–113.6

The lowest energy barrier for abstraction on a conjugated cyclic ketone was calculated to be for reaction on the methyl group of 2-methyl-cyclopentenone. The lower TS could be due to the proximity of the methyl group to the carbonyl group. Combined with a more facile addition onto the carbon double bond, this is in agreement with a higher overall rate coefficient observed at room temperature. Abstraction on the methyl group from 3-methyl-cyclopentenone is expected to be less favorable than in the case of 2-methyl-cyclopentenone. The higher TS is likely due to the methyl group being further away from the carbonyl.

Abstraction product distributions are likely to follow the height of the energy barriers^25^ at room temperature and, as the temperature increases, the product exothermicity. The radical formed by abstraction on the methyl group is expected to be the main 2-methyl-cyclopentenone abstraction product. The high-temperature reaction of OH with cyclopentenone will likely be a distribution between the α and β radical isomers. Abstraction on the methyl group of 3-methyl-cyclopentenone is expected to be the least favored abstraction channel. Instead, the product distribution should be dominated by abstraction in α and β position.

All reactions of OH with cyclic conjugated ketone investigated here are expected to lead to the formation of resonance-stabilized and/or vinoxy-like radicals, either through association or abstraction mechanisms. In the atmosphere, the OH containing vinoxy radicals are expected to be the main reaction products. Further reaction with molecular oxygen may lead to the formation of peroxy radicals, propagating the oxidation chemical scheme. As these reactions are expected to be relatively slow, the substituted vinoxy radicals may also react with other abundant atmospheric compounds. In combustion, the use of cyclopentanone as a fuel additive could promote the formation of resonance-stabilized radicals through the formation of cyclopentenone and successive OH reactions. These radicals may resist oxidation by molecular oxygen and accumulate. Their self- and cross-reactions may lead to the formation of larger oxygenated hydrocarbons.

## 7 Conclusion

We found that the rate coefficients for reactions of OH with cyclopentenone and 2-methyl-cyclopentenone were above 1 × 10^–11^ cm^3^ s^–1^ at room temperature, decreasing over the 300–500 K temperature range. These trends are similar to those observed for conjugated ketone such as methyl vinyl ketone and ethyl vinyl ketone. At room temperature, addition onto the C=C unsaturation is expected to be the dominant reaction pathway forming vinoxy-like radical products. As the temperature increases, abstraction pathways become more competitive, with abstraction on the methyl group of 2-methyl-cyclopentenone and β-position of cyclopentenone being the main pathways. The reaction with 3-methyl-cyclopentenone was unexpectedly slow, with rate coefficients characteristic of an abstraction mechanism over the whole experimental temperature range. The slower reaction could be due to a combination of higher energy barriers and steric effects.

The present study provides valuable information about the reactivity of conjugated cyclic ketones in gas phase environments. Specifically, it sheds light on the preferred addition and abstraction mechanisms, as well as the effects of resonance stabilization on reaction product distributions. The relatively low-rate coefficient for the reaction with 3-methyl-cyclpentenone compared to that of 2-methyl-cyclopentenone suggests that large uncertainties about their reaction mechanisms remain. Product detection experiments and higher-level calculations are required to fully unravel the reaction mechanisms.

## Supporting Information Available

T1 diagnostic values, vibrational frequencies, rotational constants, and atomic coordinates of the reactants and intermediates. Vapor pressure measurements of the reactants and pressure dependence of the reaction rate coefficient. MESMER results at higher temperatures and for the methyl-substituted reactants (PDF)
