# Supplementary material for: Kinetic Study of OH Radical Reactions with Cyclopentenone Derivatives
Source: J Phys Chem A. 2024 Sep 17;128(38):8209–19. doi: 10.1021/acs.jpca.4c04060 (PMC11440604; doi:10.1021/acs.jpca.4c04060)
Supplement: Supplementary file 1 — jp4c04060_si_001.pdf [file jp4c04060_si_001.pdf]

Supporting Information

Kinetic Study of OH Radical Reactions with Cyclopentenone Derivatives

Patrick Rutto,[1] *Emmanuel Ubana,*[1] *Talitha M. Selby,*[2] *and Fabien Goulay*[1*]

[1]C. Eugene Bennett Department of Chemistry, West Virginia University, Morgantown, West Virginia 26506, USA

[2]Department of Mathematics and Natural Sciences, University of Wisconsin-Milwaukee, West Bend, Wisconsin 53095, USA

***Corresponding Author**: Fabien.Goulay@mail.wvu.edu

Table S1. T1 Diagnostic of OH + 2-cyclopentene-1-one

Molecule	T1 Diagnostic
CPD	0.01395296
VDW	0.01510094
TS1a	0.02767189
TS2a	0.02858390
INT1a	0.02086276
INT2a	0.01406205
TS5a	0.02335504
P2a	0.01442317
TS3a	0.01848220
TS4a	0.02450944
P1a	0.01408377
INT3a	0.01934598
TS6a	0.02386053
P3a	0.01331907

Table S2. T1 Diagnostic of 2-methyl-2-cyclopentene-1-one

Molecule	T1 Diagnostic
2MCPD	0.01313080
VDW	0.01429183
TS1b	0.01871492
TS2b	0.02706607
INT1b	0.01883838
INT2b	0.01363375
TS4b	0.01602378
TS5b	0.01957959
TS8b	0.02241703
P2b	0.01382316
INT5b	0.01653001
INT4b	0.01384231
TS7b	0.02339692
P1b	0.01409718
TS6b	0.02804480
INT6b	0.01489747

Table S3. Optimized geometries, rotational Constants, vibrational Frequencies, and ZPE-corrected energies of the OH + 2-cyclopentene-1-one stationary points calculated at the CCSD(T)/cc-pVTZ//M06-2X/6-311++G** level of theory.

Formula	Label	Optimized geometry coordinates	CCSD(T) Energy (kJ mol^{-1})	Rotational Constants (cm^{-1})	Vibrational frequencies (cm^{-1})
OH	OH	O -1.0545360 -0.0259970 0.0000000<br>H -0.0825170 -0.0259970 0.0000000	0.00	18.8113	3787.7
C_5H_6O	CPD	C 1.261214 0.875425 0.000007<br>C 0.025009 1.228645 0.000195<br>C 0.881929 0.016224 0.000023<br>C 0.049631 -1.197633 0.000353<br>C 1.472000 -0.619273 -0.000334<br>H 2.094968 1.568280 0.000015<br>H 0.432254 2.230239 0.000370<br>H -0.167270 -1.805674 0.880017<br>H -0.167853 -1.807073 -0.878163<br>H 2.050528 -0.923696 0.875795<br>H 2.049534 -0.923429 -0.877179<br>O -2.085386 -0.019871 -0.000290	0.00	0.248 0.120<br>0.084	83.30 289.66 469.86<br>537.10 635.25 763.93<br>771.54 827.66 837.20<br>932.53 998.93 1024.40<br>1034.52 1107.93<br>1166.49 1196.70<br>1241.38 1269.69<br>1334.07 1370.11<br>1452.84 1488.49<br>1678.17 1854.43<br>3075.37 3101.39<br>3107.14 3147.24<br>3212.09 3253.65
$C_5H_7O_2$	vdW	C 1.294719 -0.956815 -0.360333<br>C 1.172732 -0.228226 0.952920<br>C -0.078346 0.154549 1.230821<br>C -0.983797 -0.250636 0.124351<br>C -0.145541 -1.029026 -0.886898<br>H 1.950010 -0.395653 -1.031164<br>H 1.747104 -1.940907 -0.213922<br>H 2.033156 -0.037858 1.58411<br>H -0.422274 0.692640 2.104131<br>H -0.526878 -2.050973 -0.938207<br>H -0.267796 -0.575124 -1.870089<br>O -2.157598 0.007063 0.028115<br>O 0.668856 1.968864 -0.727232<br>H -0.161995 2.361387 -0.407104	-9.01	0.103 0.083<br>0.069	32.29 94.14 134.51<br>141.46 200.69 285.22<br>410.16 470.17 537.34<br>638.46 763.90 769.02<br>826.33 839.25 933.32<br>991.59 1024.37 1034.89<br>1105.20 1172.36<br>1196.41 1243.39<br>1274.39 1336.43<br>1369.88 1443.55<br>1479.12 1661.83<br>1846.86 3079.25<br>3101.22 3115.77<br>3156.02 3213.93<br>3248.58 3777.75
$C_5H_7O_2$	TS1a	C 1.135890 -0.157524 0.018232<br>C 0.075540 -0.817599 0.818216<br>C -1.023798 -0.021749 0.860670<br>C -0.777667 1.328148 0.235447<br>C 0.558600 1.156834 -0.500390<br>H 0.196460 -1.793715 1.269016<br>H -1.920305 -0.234927 1.427940<br>H -1.602131 1.606051 -0.420370<br>H -0.711945 2.079407 1.028230<br>H 0.403042 1.040379 -1.574945<br>H 1.270136 1.967155 -0.344080<br>O 2.244112 -0.583989 -0.186303<br>O -1.725817 -0.884968 -0.953603<br>H -1.593011 -1.841357 -0.859594	-3.05	0.134 0.076<br>0.065	-373.63, 87.77 118.89<br>198.31 235.60 321.19<br>468.48 528.75 640.13<br>699.04 755.46 797.14<br>816.82 846.96 934.29<br>976.51 1017.14 1024.46<br>1096.51 1165.73<br>1193.72 1241.19<br>1281.50 1337.21<br>1367.25 1447.21<br>1482.68 1561.90<br>1840.37 3077.08<br>3101.84 3142.60<br>3157.55 3233.20<br>3251.53 3809.54
$C_5H_7O_2$	TS2a	C 1.231230 -1.173316 -0.090505<br>C 1.260585 0.044254 0.791354<br>C 0.064774 0.690926 0.827046	5.56	0.114 0.092<br>0.069	-406.05, 107.61 124.27<br>154.80 184.56 279.38<br>469.19 527.47 647.91

		C -0.967540 -0.156590 0.146938<br>C -0.208336 -1.230257 -0.625783<br>H 1.974008 -1.080557 -0.886622<br>H 1.495833 -2.063447 0.488729<br>H 2.159134 0.373363 1.299459<br>H -0.204565 1.529891 1.453207<br>H -0.694712 -2.196856 -0.499629<br>H -0.254503 -0.955775 -1.681266<br>O -2.156680 -0.026058 0.224680<br>O 0.435380 1.649882 -0.955763<br>H 1.010929 2.352695 -0.619517			745.34 751.17 759.19<br>830.37 852.17 931.76<br>982.16 1012.14 1022.91<br>1094.37 1165.31<br>1189.16 1241.38<br>1277.41 1333.91<br>1366.44 1446.42<br>1476.45 1562.42<br>1879.92 3068.45<br>3104.09 3108.72<br>3161.27 3217.55<br>3257.39 3825.81
$C_5H_7O_2$	INT1a	C 1.196435 -0.174224 -0.014034<br>C 0.124554 -1.010073 0.496550<br>C -1.174661 -0.263293 0.467642<br>C -0.735663 1.209289 0.412000<br>C 0.606893 1.198130 -0.334196<br>H 0.257568 -2.051558 0.766399<br>H -1.820678 -0.477304 1.327467<br>H -1.506115 1.812821 -0.071936<br>H -0.596801 1.577835 1.433243<br>H 0.461806 1.257419 -1.418313<br>H 1.295186 1.992776 -0.040689<br>O 2.360572 -0.516896 -0.169335<br>O -1.875299 -0.532722 -0.750470<br>H -2.078504 -1.474014 -0.805505	-115.73	0.168 0.074 0.061	113.00 146.86 292.33<br>377.02 410.93 466.15<br>469.34 615.47 671.16<br>769.03 799.68 864.88<br>900.46 969.48 987.97<br>1046.68 1067.45<br>1158.50 1199.40<br>1214.54 1241.78<br>1305.58 1322.58<br>1347.86 1371.94<br>1401.59 1464.25<br>1492.03 1723.35<br>3073.65 3097.24<br>3099.74 3158.38<br>3166.24 3247.48<br>3884.55
$C_5H_7O_2$	INT2a	C -1.762912 -0.493561 -0.263101<br>C -0.577892 -1.372531 0.019786<br>C 0.623504 -0.598658 0.433627<br>C 0.202457 0.841057 0.112801<br>C -1.309631 0.926074 0.136236<br>H -2.022840 -0.525515 -1.328744<br>H -2.658121 -0.811664 0.278971<br>H -0.568128 -2.444057 -0.114320<br>H 0.782160 -0.659190 1.527196<br>H -1.613245 1.159029 1.162041<br>H -1.665089 1.725478 -0.511655<br>O 0.988339 1.699611 -0.180334<br>O 1.806508 -0.962159 -0.234705<br>H 2.333331 -0.157977 -0.329268	-91.03	0.127 0.100 0.060	71.69 109.84 255.02<br>309.97 350.89 419.15<br>472.27 581.50 644.40<br>685.97 774.57 832.84<br>923.81 969.99 1004.03<br>1065.66 1082.41<br>1148.05 1193.03<br>1199.63 1234.00<br>1273.66 1315.80<br>1329.01 1379.41<br>1407.12 1458.45<br>1488.03 1885.43<br>2928.42 3029.43<br>3071.13 3082.08<br>3158.83 3249.25<br>3819.42
$C_5H_7O_2$	TS5a	C 1.252681 -0.270250 -0.014647<br>C 0.007546 -1.041674 -0.004207<br>C -1.048175 -0.174183 0.040654<br>C -0.630142 1.261739 -0.157430<br>C 0.880797 1.212391 0.093821<br>H -0.033978 -2.122186 0.008043<br>H -0.962270 -0.048807 1.840097<br>H -0.864738 1.534951 -1.190358<br>H -1.178174 1.934298 0.501790<br>H 1.474331 1.807269 -0.598804<br>H 1.122779 1.534373 1.110825<br>O 2.377847 -0.704103 -0.082141	17.42	0.193 0.069 0.053	-1000.74, 137.13 183.83<br>349.21 441.21 492.43<br>523.49 526.06 555.80<br>593.56 625.91 647.66<br>801.70 841.90 863.07<br>922.26 1013.67 1022.80<br>1153.16 1161.64<br>1219.99 1220.93<br>1254.80 1287.60<br>1325.98 1442.31<br>1457.74 1491.54<br>1603.07 1825.62

		O -2.356041 -0.442824 -0.089906<br>H -2.508643 -1.392626 -0.044362			3081.92 3086.00<br>3144.63 3158.87<br>3247.56 3873.47
$C_5H_6O_2$	P2a	C 1.232865 -0.273129 -0.000010<br>C -0.017855 -1.041438 0.000051<br>C -1.054952 -0.186624 0.000010<br>C -0.664248 1.262234 0.000012<br>C 0.869191 1.217608 -0.000022<br>H -0.055109 -2.122094 0.000142<br>H -1.084730 1.755829 -0.879334<br>H -1.084593 1.755664 0.879536<br>H 1.311478 1.686541 -0.879843<br>H 1.311436 1.686479 0.879858<br>O 2.358663 -0.706469 -0.000027<br>O -2.367441 -0.447926 -0.000025<br>H -2.518262 -1.399170 -0.000189	-12.25	0.201 0.070 0.053	97.67 171.10 360.39<br>461.23 509.77 535.72<br>567.34 590.36 636.37<br>824.24 855.66 855.82<br>919.13 1018.39 1023.60<br>1158.14 1169.50<br>1225.10 1238.58<br>1246.02 1284.96<br>1331.40 1450.72<br>1460.98 1491.85<br>1698.28 1844.77<br>3083.40 3103.73<br>3120.80 3150.92<br>3247.45 3873.91
$C_5H_7O_2$	TS3a	C -1.812272 -0.377870 -0.213162<br>C -0.699553 -1.363570 0.054849<br>C 0.556395 -0.596869 0.118799<br>C 0.286411 0.831272 0.025148<br>C -1.225402 1.011259 0.136976<br>H -2.048934 -0.410185 -1.285218<br>H -2.729626 -0.599111 0.331546<br>H -0.734742 -2.438760 -0.015165<br>H 0.001054 -0.880181 1.195735<br>H -1.470096 1.310222 1.161628<br>H -1.562105 1.805092 -0.527683<br>O 1.148786 1.682213 -0.098181<br>O 1.789042 -1.112517 -0.096466<br>H 2.408345 -0.369980 -0.039321	39.95	0.127 0.100 0.058	-1888.51, 152.62 214.24<br>251.33 332.39 454.89<br>463.84 491.83 597.20<br>622.15 672.37 769.59<br>838.95 921.75 994.18<br>1021.87 1071.91<br>1138.23 1182.74<br>1202.79 1229.31<br>1260.47 1301.04<br>1324.23 1440.44<br>1468.90 1487.59<br>1499.17 1776.18<br>2192.80 3018.41<br>3067.04 3130.68<br>3154.87 3280.26<br>3790.52
$C_5H_7O_2$	TS4a	C -1.815138 -0.448607 -0.013739<br>C -0.607060 -1.340363 -0.131015<br>C 0.537414 -0.634211 0.053013<br>C 0.247981 0.828158 -0.005676<br>C -1.257837 0.991183 0.003746<br>H -2.506862 -0.609532 -0.843392<br>H -2.365971 -0.674430 0.905640<br>H -0.660189 -2.413584 -0.250642<br>H 0.344531 -0.357650 1.848807<br>H -1.555149 1.561930 0.884707<br>H -1.552503 1.566404 -0.875614<br>O 1.108901 1.666683 -0.087366<br>O 1.802759 -1.063094 -0.049523<br>H 2.370700 -0.278815 -0.012366	21.80	0.129 0.100 0.059	-943.33, 104.18 213.53<br>281.02 392.48 438.12<br>494.81 510.29 523.35<br>618.21 641.79 705.73<br>776.79 792.54 859.87<br>934.02 1008.39 1036.06<br>1124.79 1165.56<br>1217.11 1236.63<br>1257.26 1316.73<br>1335.05 1431.56<br>1450.51 1490.53<br>1662.68 1854.70<br>3057.19 3100.71<br>3105.78 3147.80<br>3246.93 3781.03
$C_5H_6O_2$	P1a	C -1.815061 -0.441498 -0.000021<br>C -0.603299 -1.342725 -0.000273<br>C 0.535725 -0.644468 0.000116<br>C 0.258458 0.809022 0.000101<br>C -1.244582 0.991154 0.000225<br>H -2.439608 -0.628496 -0.877168<br>H -2.439692 -0.628820 0.876974<br>H -0.669884 -2.422770 -0.000765	-3.33	0.134 0.102 0.059	93.70 226.62 289.59<br>368.36 507.44 514.05<br>622.66 632.10 708.73<br>796.80 804.32 881.37<br>936.21 1019.42 1037.61<br>1135.52 1167.37<br>1223.35 1235.40<br>1266.66 1321.27

		H -1.536316 1.568253 0.879419<br>H -1.536270 1.568481 -0.878866<br>O 1.127212 1.648695 -0.000259<br>O 1.806006 -1.073743 0.000338<br>H 2.368583 -0.285172 -0.001111			1338.21 1443.98<br>1453.73 1500.72<br>1761.18 1843.69<br>3073.77 3099.40<br>3104.46 3144.01<br>3239.12 3784.66
$C_5H_7O_2$	INT3a	C -1.817708 -0.160158 -0.205632<br>C -0.829354 -1.309301 0.124795<br>C 0.482192 -0.628154 0.005317<br>C 0.386793 0.793918 0.007274<br>C -1.083045 1.154958 0.133632<br>H -2.038432 -0.183429 -1.274309<br>H -2.759899 -0.265902 0.330386<br>H -0.922401 -2.164602 -0.548167<br>H -0.969455 -1.684304 1.146729<br>H -1.274238 1.472220 1.163915<br>H -1.347097 1.985342 -0.521268<br>O 1.368814 1.535465 -0.046940<br>O 1.649794 -1.249575 -0.029662<br>H 2.329393 -0.554025 -0.076774	-176.57	0.128 0.100 0.058	154.60 169.08 282.46<br>290.51 475.83 568.29<br>600.01 640.64 659.04<br>746.62 841.73 876.36<br>921.31 1024.33 1028.50<br>1077.36 1132.01<br>1200.90 1223.19<br>1249.54 1304.36<br>1311.29 1340.31<br>1470.22 1472.39<br>1481.07 1510.16<br>1560.27 1682.79<br>3034.32 3069.60<br>3092.30 3107.53<br>3137.68 3150.07<br>3713.71
$C_5H_7O_2$	TS6a	C 1.677726 -0.509549 0.115434<br>C 0.639416 -1.344456 -0.093957<br>C -0.616433 -0.600551 -0.439657<br>C -0.214406 0.848840 -0.131929<br>C 1.303476 0.937781 -0.131500<br>H 1.704167 -0.339497 2.035999<br>H 2.703897 -0.831065 0.239415<br>H 0.673866 -2.423351 -0.026169<br>H -0.809638 -0.671047 -1.523964<br>H 1.646908 1.279761 -1.114491<br>H 1.659217 1.645077 0.617880<br>O -1.746675 -1.007863 0.283892<br>H -2.303422 -0.228668 0.405986	64.35	0.124 0.103 0.062	-794.94, 86.02 207.83<br>249.66 335.25 387.90<br>440.75 456.12 503.06<br>589.95 685.78 746.43<br>774.84 805.49 959.98<br>965.91 1011.24 1018.23<br>1091.70 1127.21<br>1173.37 1206.34<br>1221.41 1287.43<br>1332.26 1372.26<br>1412.60 1456.82<br>1617.32 1895.58<br>2971.40 3063.35<br>3139.68 3222.96<br>3247.80 3831.07
$C_5H_6O_2$	P3a	C -1.696845 -0.554643 -0.179547<br>C -0.652652 -1.361448 0.007676<br>C 0.577905 -0.601998 0.417630<br>C 0.163574 0.843903 0.111573<br>C -1.353901 0.903132 0.009439<br>H -2.693324 -0.887644 -0.440317<br>H -0.660755 -2.439773 -0.080542<br>H 0.733182 -0.688544 1.505825<br>H -1.769602 1.318442 0.934358<br>H -1.663665 1.554544 -0.810209<br>O 0.950304 1.723729 -0.099287<br>O 1.739811 -0.977906 -0.273430<br>H 2.304757 -0.197288 -0.327994	47.04	0.129 0.108 0.062	81.58 244.20 274.10<br>328.46 437.46 494.07<br>589.60 676.88 719.92<br>773.37 793.61 961.93<br>978.60 999.61 1016.88<br>1096.52 1135.59<br>1173.71 1206.90<br>1224.85 1291.34<br>1341.14 1375.13<br>1418.45 1453.89<br>1697.13 1894.56<br>2983.31 3063.84<br>3121.68 3224.26<br>3247.36 3836.03

Table S4. Optimized geometries, rotational Constants, vibrational Frequencies, and ZPE-corrected energies of the OH + 2-methyl-2-cyclopentene-1-one stationary points calculated at the CCSD(T)/cc-pVTZ//M06-2X/6-311++G** level of theory.

Formula	Label	Optimized geometry coordinates	CCSD(T) Energy (kJ mol^{-1})	Rotational Constants (cm^{-1})	Vibrational frequencies (cm^{-1})
OH	OH	O -1.0545360 -0.0259970 0.0000000<br>H -0.0825170 -0.0259970 0.0000000	0.00	18.8113	3787.7
C_6H_8O	2MCP	C 0.348437 -1.397973 0.000052<br>C -0.641997 -0.500621 -0.000021<br>C -0.053135 0.866762 0.000030<br>C 1.466457 0.726757 -0.000259<br>C 1.728139 -0.786782 0.000164<br>H 0.200154 -2.472920 0.000158<br>H 1.868182 1.232993 -0.879588<br>H 1.868850 1.233850 0.878238<br>H 2.294607 -1.112503 -0.876249<br>H 2.294297 -1.112088 0.876941<br>O -0.673681 1.900393 0.000208<br>C -2.119436 -0.701834 -0.000091<br>H -2.378584 -1.760676 0.000367<br>H -2.564527 -0.224398 0.875926<br>H -2.564325 -0.225257 -0.876704	0.00	0.128<br>0.099<br>0.057	81.50 134.03 224.29<br>255.39 327.88 490.83<br>587.24 608.65 695.83<br>775.33 808.14 874.81<br>930.73 998.73 1026.89<br>1050.28 1069.53<br>1093.08 1165.00<br>1224.71 1238.94<br>1293.34 1332.48<br>1361.13 1420.04<br>1453.83 1477.25<br>1492.13 1493.07<br>1733.51 1846.70<br>3067.11 3073.21<br>3099.90 3104.04<br>3128.13 3144.85<br>3156.92 3203.29
$C_6H_9O_2$	vdW	C -0.394118 -0.985610 -0.910416<br>C 0.669342 -0.293103 -0.476385<br>C 0.201447 1.012649 0.070481<br>C -1.314416 1.066972 -0.069720<br>C -1.702162 -0.255879 -0.747202<br>H -0.341635 -1.978810 -1.343965<br>H -1.746877 1.168117 0.926446<br>H -1.590742 1.950526 -0.647368<br>H -2.389073 -0.848129 -0.138985<br>H -2.177527 -0.104945 -1.720305<br>O 0.912245 1.861145 0.549279<br>C 2.116360 -0.654680 -0.483069<br>H 2.670904 0.028206 -1.131480<br>H 2.544773 -0.539966 0.516364<br>H 2.273211 -1.676809 -0.828007<br>O -0.547901 -1.364552 1.710938<br>H 0.383504 -1.313021 1.983429	-9.30	0.074<br>0.065<br>0.053	40.72 85.86 105.29<br>117.59 136.37 168.30<br>237.20 253.99 326.37<br>442.70 490.20 585.93<br>609.31 696.74 774.50<br>807.36 876.18 929.87<br>996.31 1026.95 1049.19<br>1063.05 1094.01<br>1172.99 1224.29<br>1242.95 1295.92<br>1336.27 1357.96<br>1416.56 1447.86<br>1473.10 1484.92<br>1491.53 1712.00<br>1844.11 3063.25<br>3077.09 3103.94<br>3116.96 3120.64<br>3152.57 3156.52<br>3207.74 3793.31
$C_6H_9O_2$	INT 1b	C -1.103601 -0.245599 0.353775<br>H -1.285660 -0.286290 1.442427<br>C -0.581582 -1.609197 -0.141622<br>H -1.077028 -2.451047 0.344339<br>C 0.931781 -1.554054 0.107696<br>H 1.524870 -2.147911 -0.587152<br>C 0.038404 0.683114 0.102247<br>C 1.265672 -0.068332 -0.018921<br>O 2.380261 0.404179 -0.178240<br>C -0.071975 2.153719 0.044629	-124.75	0.097<br>0.067<br>0.042	99.04 121.94 156.50<br>199.09 221.05 253.52<br>338.50 480.37 513.92<br>539.25 569.04 654.08<br>711.85 834.64 899.36<br>965.47 992.60 1007.69<br>1037.22 1087.49<br>1132.05 1158.92<br>1205.44 1221.06<br>1277.06 1301.20

		O -2.265825 0.220218 -0.304161<br>H 1.187323 -1.876712 1.122369<br>H -0.783478 -1.660183 -1.214660<br>H -0.807755 2.447644 -0.709513<br>H -0.431316 2.550626 1.001418<br>H 0.898120 2.594589 -0.184588<br>H -3.012758 -0.323804 -0.042255			1316.53 1330.34<br>1381.17 1404.49<br>1458.29 1469.64<br>1471.20 1487.81<br>1501.95 1696.33<br>2954.10 3039.27<br>3071.30 3081.73<br>3097.18 3134.45<br>3147.73 3160.00<br>3902.43
$C_6H_9O_2$	INT 2b	C -0.441103 -1.374426 0.332448<br>C 0.628215 -0.373391 0.050963<br>C -0.176937 0.933840 -0.049929<br>C -1.625078 0.610625 -0.357813<br>C -1.824355 -0.800479 0.230273<br>H -0.221288 -2.376930 0.671488<br>H -2.287619 1.377456 0.040349<br>H -1.736692 0.600441 -1.447133<br>H -2.290833 -0.736383 1.221872<br>H -2.489072 -1.417409 -0.380954<br>O 0.310365 2.013826 0.146002<br>C 1.353956 -0.624807 -1.279956<br>H 0.651300 -0.683099 -2.114833<br>H 2.065335 0.182987 -1.470638<br>H 1.896682 -1.568862 -1.210045<br>O 1.565844 -0.290890 1.107550<br>H 1.914323 0.610129 1.105557	-89.63	0.090<br>0.073<br>0.055	50.93 111.98 210.65<br>253.88 297.98 304.40<br>356.60 377.06 459.34<br>496.44 562.90 631.97<br>696.84 725.52 806.00<br>904.80 952.12 978.52<br>981.36 1039.52 1085.70<br>1089.39 1149.37<br>1165.63 1213.51<br>1235.35 1283.09<br>1328.39 1362.15<br>1389.40 1408.74<br>1457.72 1486.68<br>1491.02 1492.14<br>1882.11 3024.93<br>3056.14 3071.53<br>3083.04 3135.26<br>3149.56 3157.57<br>3241.22 3821.28
$C_6H_9O_2$	INT 3b	C -1.155416 0.535544 0.006931<br>H -1.711389 1.413874 0.350526<br>C -1.019167 -0.544144 1.095390<br>H -0.757437 -0.071660 2.045934<br>C 0.128924 -1.425675 0.594975<br>H -0.227834 -2.199655 -0.089443<br>C 0.285869 0.875110 -0.358632<br>C 1.008161 -0.486483 -0.225765<br>O 2.088252 -0.739811 -0.677028<br>C 0.928061 1.817213 0.596960<br>O -1.742818 -0.002383 -1.168971<br>H 0.714158 -1.910920 1.375771<br>H -1.953955 -1.088780 1.243287<br>H 0.373275 1.218022 -1.394179<br>H 0.405975 2.167086 1.477669<br>H 1.966471 2.085410 0.464085<br>H -2.631321 -0.305212 -0.964805	-76.45	0.093<br>0.066<br>0.055	74.94 124.56 179.31<br>215.21 255.75 309.82<br>398.37 464.52 472.81<br>484.30 542.54 626.97<br>723.15 797.63 818.46<br>877.78 923.39 987.17<br>1026.04 1049.69<br>1099.05 1107.12<br>1134.72 1184.92<br>1190.95 1235.05<br>1246.85 1299.24<br>1305.07 1339.33<br>1366.13 1430.89<br>1451.98 1458.43<br>1496.17 1879.62<br>3063.49 3077.82<br>3082.26 3092.71<br>3124.74 3152.39<br>3177.22 3290.13<br>3898.69
$C_6H_9O_2$	INT 4b	C -1.196559 0.563221 0.018338<br>H -1.640383 1.459459 0.468764<br>C -1.146182 -0.648367 0.979231<br>H -0.864983 -0.292048 1.974085<br>C -0.065521 -1.554979 0.391626<br>H -0.469703 -2.172663 -0.415471<br>C 0.297505 0.799472 -0.357005	-62.12	0.091<br>0.068<br>0.053	58.42 181.35 203.23<br>233.39 260.55 272.93<br>446.90 474.25 515.33<br>639.36 681.42 775.84<br>815.40 860.74 909.75<br>957.43 1000.52 1003.89<br>1029.65 1041.08

		C 0.930676 -0.590412 -0.233726<br>O 2.059585 -0.840854 -0.552594<br>C 0.982480 1.782354 0.594497<br>O -1.811835 0.216840 -1.153202<br>H 0.430249 -2.211768 1.105398<br>H -2.124381 -1.122029 1.055662<br>H 0.564205 2.783484 0.482379<br>H 0.865120 1.473584 1.636319<br>H 2.049082 1.817680 0.368487<br>H 0.394396 1.148671 -1.387016			1130.78 1143.19<br>1189.17 1210.11<br>1246.97 1276.42<br>1302.53 1313.98<br>1338.80 1377.85<br>1403.48 1452.92<br>1493.38 1500.15<br>1505.27 1882.14<br>3041.75 3066.09<br>3084.44 3088.28<br>3108.49 3142.38<br>3147.55 3156.06<br>3156.88
$C_6H_9O_2$	INT 5b	C 1.067594 -0.894624 -0.179247<br>H 0.409048 -1.730369 -0.391790<br>C -1.980889 -1.437628 0.640779<br>H -2.336076 -2.444014 0.465190<br>C -1.817408 -0.482766 -0.494991<br>H -2.764783 0.020511 -0.724111<br>C 0.613193 0.356136 -0.032249<br>C -0.832415 0.643832 -0.177744<br>O -1.240800 1.776164 -0.033272<br>C 1.508361 1.518843 0.280450<br>O 2.382299 -1.191868 -0.058893<br>H -1.509788 -1.009416 -1.403780<br>H -1.971838 -1.070379 1.658354<br>H 1.186100 2.001730 1.205112<br>H 2.543500 1.198081 0.381746<br>H 1.438895 2.271308 -0.507763<br>H 2.522338 -2.134574 -0.167622	-32.85	0.090<br>0.054<br>0.036	69.46 89.87 99.58<br>148.22 172.83 225.82<br>274.99 294.68 312.19<br>412.09 455.61 485.79<br>501.17 599.46 734.46<br>774.40 833.35 923.34<br>974.85 1034.46 1059.87<br>1077.00 1112.89<br>1189.73 1214.95<br>1272.90 1321.90<br>1366.42 1402.61<br>1442.82 1464.47<br>1472.78 1485.30<br>1491.91 1756.73<br>1796.86 3034.25<br>3075.12 3088.69<br>3133.31 3170.91<br>3176.08 3201.11<br>3278.72 3927.18
$C_6H_9O_2$	TS1b	C -1.164448 0.255538 0.370285<br>H -1.659969 0.749895 1.212147<br>C -1.084101 -1.269730 0.565103<br>H -1.002411 -1.486085 1.633381<br>C 0.200876 -1.702187 -0.153958<br>H 0.013839 -1.922515 -1.208971<br>C 0.267640 0.677857 0.231686<br>C 1.094772 -0.467815 -0.090243<br>O 2.296199 -0.435889 -0.298595<br>C 0.746958 2.075434 0.244070<br>O -1.853147 0.567635 -0.847428<br>H 0.696550 -2.566252 0.287397<br>H -1.984787 -1.749872 0.183940<br>H 0.261992 2.645028 -0.556995<br>H 0.497585 2.571547 1.188066<br>H 1.826555 2.104830 0.094566<br>H -2.563953 1.184871 -0.667002	-6.85	0.090<br>0.071<br>0.046	-371.58, 84.14 115.82<br>139.53 186.81 260.16<br>379.26 439.61 483.38<br>574.61 620.57 671.30<br>763.77 809.51 894.49<br>942.82 982.40 998.17<br>1050.35 1061.11<br>1079.87 1163.85<br>1207.46 1232.07<br>1260.81 1302.10<br>1311.32 1343.92<br>1385.82 1410.46<br>1443.54 1465.83<br>1478.66 1486.13<br>1488.23 1704.17<br>3037.38 3061.58<br>3083.22 3086.87<br>3089.91 3145.14<br>3152.74 3153.80<br>3929.54
$C_6H_9O_2$	TS2b	C -0.319896 -1.222639 -0.677230<br>C 0.611550 -0.281312 -0.347597<br>C -0.115636 0.997990 -0.035247<br>C -1.589758 0.657669 0.124463	-0.28	0.082<br>0.072<br>0.055	-258.66, 103.77 132.39<br>147.83 160.32 202.36<br>241.47 263.71 308.18<br>487.06 545.98 622.02

		C -1.731365 -0.770581 -0.426678<br>H -0.080969 -2.213769 -1.047601<br>H -1.814140 0.699165 1.191789<br>H -2.205063 1.397501 -0.386678<br>H -2.227567 -1.443969 0.276403<br>H -2.307946 -0.799307 -1.356640<br>O 0.396550 2.080815 0.038386<br>C 2.080326 -0.278815 -0.596098<br>H 2.295040 0.276577 -1.512795<br>H 2.586469 0.231466 0.223561<br>H 2.473461 -1.291218 -0.698812<br>O 0.448324 -0.807362 1.643673<br>H 0.910400 -1.657943 1.604626			694.17 740.28 764.88<br>782.75 851.17 925.60<br>986.18 1011.37 1038.27<br>1051.14 1097.69<br>1165.45 1220.57<br>1244.41 1295.57<br>1335.03 1351.04<br>1412.21 1446.07<br>1476.17 1478.25<br>1486.18 1613.86<br>1873.74 3066.12<br>3070.00 3103.36<br>3107.85 3138.84<br>3159.80 3164.40<br>3206.51 3822.77
$C_6H_9O_2$	TS3b	C -1.168794 0.267840 0.365871<br>H -1.616308 0.766896 1.232740<br>C -1.024085 -1.252431 0.596875<br>H -0.902238 -1.433659 1.667855<br>C 0.248261 -1.673493 -0.147888<br>H 0.042640 -1.908314 -1.196674<br>C 0.247380 0.720670 0.133889<br>C 1.127709 -0.428442 -0.106868<br>O 2.328890 -0.377960 -0.273552<br>C 0.744633 2.083413 0.331753<br>O -1.925069 0.581147 -0.803665<br>H 0.764669 -2.525185 0.293580<br>H -1.918811 -1.783579 0.266766<br>H 0.330947 1.459908 -0.840203<br>H 0.055448 2.884735 0.551988<br>H 1.808141 2.257015 0.249255<br>H -2.845675 0.351344 -0.649364	51.98	0.094<br>0.069<br>0.045	-1631.66, 76.08 144.32<br>215.63 262.25 297.88<br>374.71 420.20 478.19<br>500.75 589.84 633.58<br>665.08 699.11 793.88<br>814.55 885.22 933.40<br>994.72 1048.72 1055.78<br>1099.85 1146.21<br>1178.96 1208.04<br>1228.98 1281.28<br>1305.27 1326.19<br>1348.21 1389.59<br>1425.74 1453.32<br>1466.43 1490.45<br>1782.73 2314.52<br>3047.82 3078.79<br>3080.51 3127.64<br>3150.15 3190.91<br>3318.22 3887.56
$C_6H_9O_2$	TS4b	C 0.899669 -0.891152 0.345651<br>H 1.057858 -1.702574 1.057084<br>C 1.612971 0.421904 0.676051<br>H 1.558816 0.595578 1.754298<br>C 0.814723 1.497184 -0.074653<br>H 1.172476 1.613104 -1.101936<br>C -0.530109 -0.554584 -0.012211<br>C -0.601730 0.940466 -0.140186<br>O -1.623763 1.565478 -0.249289<br>C -1.715874 -1.375050 .399989<br>O 1.125082 -1.310765 -1.000642<br>H 0.814851 2.478923 0.396096<br>H 2.663729 0.381150 0.388945<br>H -1.548793 -2.432619 0.189836<br>H -1.891374 -1.263624 1.474271<br>H -2.608641 -1.038381 -0.127059<br>H -0.107379 -0.901876 -1.199925	27.06	0.088<br>0.076<br>0.049	-1846.72, 113.55 166.31<br>180.76 221.66 271.44<br>382.98 461.59 486.34<br>618.23 641.17 680.24<br>804.07 828.97 891.12<br>911.45 959.39 991.89<br>1040.59 1071.69<br>1091.28 1098.72<br>1163.94 1188.89<br>1248.53 1263.24<br>1289.93 1330.16<br>1338.07 1385.66<br>1408.04 1457.76<br>1484.78 1489.48<br>1492.70 1846.66<br>1955.09 3059.10<br>3080.77 3086.90<br>3118.00 3130.25<br>3143.88 3160.23<br>3161.81
$C_6H_9O_2$	TS5b	C 1.220793 0.406104 -0.260451<br>H 1.716762 1.216796 -0.823894	24.35	0.089<br>0.064	-197.84, 93.28 117.80<br>131.63 190.01 283.02

		C 1.539577 -0.782868 -1.083642<br>H 0.398314 -1.815730 -0.837287<br>C -0.280710 -1.825950 0.086777<br>H 0.299640 -1.934633 1.002268<br>C -0.264283 0.643720 -0.204466<br>C -1.062919 -0.531419 0.074535<br>O -2.267757 -0.485034 0.276233<br>C -0.859562 1.998200 -0.293537<br>O 1.755263 0.347334 1.072780<br>H -0.928167 -2.686495 -0.073084<br>H 2.320989 -1.351574 -0.550645<br>H -0.407079 2.680086 0.436117<br>H -0.676192 2.430592 -1.283522<br>H -1.933468 1.951393 -0.117175<br>H 1.551782 1.164440 1.539829		0.046	299.41 379.16 392.15<br>449.78 465.96 543.33<br>579.42 627.17 728.29<br>831.53 919.02 950.89<br>975.43 990.26 1022.97<br>1050.27 1096.36<br>1141.87 1170.82<br>1235.81 1312.25<br>1332.33 1361.15<br>1395.39 1424.99<br>1428.62 1479.50<br>1482.83 1545.92<br>1688.19 2406.01<br>2979.16 3033.19<br>3035.71 3081.93<br>3121.20 3164.90<br>3191.04 3865.66
$C_6H_9O_2$	TS6b	C -0.907317 -1.077492 -1.249968<br>C 0.716800 -0.385623 0.024152<br>C -0.046893 0.935291 0.092363<br>C -1.527978 0.751790 0.334787<br>C -1.863217 -0.664866 -0.169069<br>H 0.250062 -1.216175 -0.915063<br>H -1.747173 0.894117 1.396262<br>H -2.050350 1.527900 -0.225253<br>H -1.844810 -1.379692 0.662773<br>H -2.862937 -0.706734 -0.607722<br>O 0.514798 1.983459 -0.075517<br>C 2.116528 -0.274647 -0.522359<br>H 2.088440 0.135516 -1.531952<br>H 2.689174 0.406801 0.110976<br>H 2.607475 -1.250421 -0.548346<br>O 0.584969 -1.022509 1.242240<br>H 1.144442 -1.805631 1.265103	8.86	0.090<br>0.069<br>0.053	- 616.70, 75.94 155.08<br>218.19 232.09 245.09<br>309.52 343.13 390.25<br>490.92 532.46 575.67<br>595.14 668.18 718.64<br>859.61 878.09 940.89<br>958.36 987.22 1037.50<br>1058.60 1090.55<br>1140.78 1201.34<br>1222.19 1261.14<br>1292.59 1328.73<br>1387.15 1409.75<br>1449.60 1466.27<br>1476.65 1488.10<br>1864.25 2216.31<br>3036.11 3065.39<br>3086.97 3109.70<br>3143.02 3146.35<br>3164.90 3871.98
$C_6H_9O_2$	TS7b	C 1.088456 -0.459088 -0.415408<br>H 0.847347 -1.188949 -1.182613<br>C -0.353810 -1.857429 0.610965<br>H -0.061901 -2.859889 0.315665<br>C -1.473821 -1.168768 -0.110310<br>H -2.448249 -1.278074 0.374702<br>C 0.271458 0.615040 -0.141713<br>C -1.164878 0.337555 -0.174193<br>O -2.029608 1.186604 -0.161247<br>C 0.779535 1.921184 0.378446<br>O 2.401539 -0.374652 -0.087717<br>H -1.582925 -1.552179 -1.131298<br>H -0.206200 -1.617058 1.658164<br>H 0.907422 1.895959 1.465962<br>H 1.744189 2.173575 -0.062952<br>H 0.050011 2.698937 0.148804<br>H 2.893214 -1.098913 -0.481444	12.18	0.091<br>0.064<br>0.041	-640.07, 97.49 109.11<br>151.61 217.50 258.82<br>296.29 349.84 441.15<br>471.95 521.02 549.09<br>605.21 665.07 734.08<br>781.44 809.67 889.05<br>976.82 1020.20 1041.88<br>1080.01 1093.52<br>1198.72 1210.14<br>1248.97 1294.44<br>1325.23 1382.48<br>1414.61 1457.63<br>1475.22 1481.16<br>1492.69 1601.50<br>1791.48 3050.05<br>3054.27 3096.87<br>3119.25 3142.00<br>3160.06 3160.77<br>3242.98 3916.41

$C_5H_6O_2$	P1b	C -0.603241 -1.342721 -0.000253<br>C 0.535763 -0.644415 0.000066<br>C 0.258449 0.809054 0.000068<br>C -1.244646 0.991072 0.000104<br>C -1.815059 -0.441553 0.000060<br>H -0.669688 -2.422767 -0.000735<br>H -1.536384 1.568354 0.879188<br>H -1.536278 1.568180 -0.879131<br>H -2.439400 -0.628788 0.877300<br>H -2.439912 -0.628672 -0.876834<br>O 1.127146 1.648781 -0.000177<br>O 1.806033 -1.073746 0.000234<br>H 2.368632 -0.285205 -0.000517	-35.16	0.134<br>0.102<br>0.059	93.64 226.64 289.55<br>368.35 507.28 514.03<br>622.65 632.09 708.72<br>796.80 804.34 881.36<br>936.23 1019.42 1037.65<br>1135.48 1167.39<br>1223.32 1235.42<br>1266.66 1321.27<br>1338.20 1443.97<br>1453.73 1500.73<br>1761.15 1843.69<br>3073.74 3099.41<br>3104.42 3144.04<br>3239.20 3784.83
$C_6H_8O_2$	P2b	C -1.031853 -0.239550 -0.000078<br>C -0.571405 -1.665834 -0.002490<br>H -0.966878 -2.186239 0.873105<br>C 0.958479 -1.539869 0.005028<br>H 1.429736 -1.989008 -0.870126<br>C -0.047416 0.677585 0.003278<br>C 1.237241 -0.034793 0.001363<br>O 2.335169 0.470035 -0.005857<br>C -0.161418 2.168125 0.002077<br>O -2.355947 -0.026713 -0.001935<br>H 1.419006 -1.982059 0.889525<br>H -0.957411 -2.179992 -0.886036<br>H -0.851734 2.527644 -0.767044<br>H -0.494380 2.553737 0.970010<br>H 0.821609 2.595007 -0.202794<br>H-2.535486 0.920336 0.000636	-11.23	0.099<br>0.070<br>0.042	28.99 100.02 148.33<br>234.50 252.25 345.12<br>450.41 539.16 563.63<br>565.89 595.36 664.18<br>704.89 839.16 909.97<br>1014.33 1015.49<br>1057.45 1074.51<br>1097.81 1166.48<br>1235.00 1236.01<br>1282.79 1309.77<br>1344.00 1401.13<br>1445.52 1462.14<br>1492.24 1496.10<br>1501.98 1738.86<br>1835.59 3048.38<br>3081.49 3102.41<br>3106.16 3118.01<br>3142.92 3149.09<br>3860.71

Table S5. Optimized geometries, rotational Constants, vibrational Frequencies, and ZPE-corrected energies of the OH + 3-methyl-2-cyclopentene-1-one stationary points calculated at the CCSD(T)/cc-pVTZ//M06-2X/6-311++G** level of theory.

Formula	Label	Optimized geometry coordinates	CCSD(T) Energy (kJ mol^{-1})	Rotational Constants (cm^{-1})	Vibrational frequencies (cm^{-1})
OH	OH	O -1.0545360 -0.0259970 0.0000000<br>H -0.0825170 -0.0259970 0.0000000	0.00	18.8113	3787.7
C_6H_8O	3MCP	C -1.034788 -0.166258 0.000133<br>C -0.006046 -1.022357 0.000263<br>C 1.278538 -0.291338 0.000068<br>C 0.951455 1.204316 0.000040<br>C -0.580305 1.277500 0.000010<br>H -0.063689 -2.102922 0.000378<br>H 1.405896 1.663474 0.879342<br>H 1.405983 1.663598 -0.879138<br>H -0.979547 1.795938 0.876882<br>H -0.979503 1.795623 -0.877120<br>O 2.387005 -0.764747 -0.000221<br>C -2.488484 -0.499898 -0.000104<br>H -2.974756 -0.067041 -0.879380<br>H -2.657032 -1.576368 0.000219<br>H -2.975617 -0.066118 0.878123		0.194<br>0.068<br>0.052	88.71 140.76 181.50<br>295.38 431.69 486.86<br>549.99 577.08 639.41<br>826.07 851.98 861.54<br>884.20 993.78 1002.42<br>1031.59 1066.07 1165.44<br>1172.08 1192.76 1240.48<br>1276.90 1311.25 1359.23<br>1416.24 1457.34 1483.00<br>1484.70 1489.00 1715.11<br>1848.12 3055.22 3065.58<br>3097.41 3099.28 3112.10<br>3144.94 3157.71 3240.79
$C_6H_9O_2$	vdW	C 0.973383 -0.273489 -0.373930<br>C -0.053923 0.318807 -1.006953<br>C -1.338541 -0.071369 -0.385251<br>C -1.017982 -1.036889 0.755559<br>C 0.508935 -1.176553 0.745030<br>H 0.012059 0.997847 -1.847892<br>H -1.395175 -0.608879 1.685113<br>H -1.543347 -1.977564 0.583031<br>H 0.966344 -0.857392 1.684892<br>H 0.841087 -2.201586 0.554361<br>O -2.437948 0.316504 -0.694399<br>C 2.424912 -0.104298 -0.658221<br>H 2.894775 -1.076097 -0.833923<br>H 2.598540 0.537655 -1.521192<br>H 2.911815 0.339305 0.215128<br>O 0.424901 1.752414 1.229025<br>H -0.162432 2.358120 0.746073	-12.13	0.094<br>0.052<br>0.048	42.81 69.28 125.39<br>131.68 141.99 175.54<br>201.65 297.45 411.53<br>460.89 488.18 545.96<br>577.96 640.98 821.80<br>852.31 854.23 885.32<br>988.21 1003.76 1033.36<br>1062.63 1167.62 1177.70<br>1191.43 1243.65 1279.26<br>1311.73 1362.70 1412.74<br>1447.02 1478.06 1481.53<br>1483.23 1689.25 1844.05<br>3058.32 3071.75 3103.57<br>3111.42 3119.21 3151.73<br>3159.33 3232.79 3789.37
$C_6H_9O_2$	INT 1C	C -1.065126 -0.175037 -0.048142<br>C 0.030029 0.699098 0.458769<br>C 1.264289 -0.103938 0.055739<br>C 0.914389 -1.579488 0.061075<br>C -0.627577 -1.613555 -0.046939<br>H 0.016786 0.741120 1.568808<br>H 1.430484 -2.080405 -0.757594<br>H 1.278108 -2.018186 0.994283<br>H -0.958114 -2.122456 -0.958159	-114.46	0.098<br>0.068<br>0.042	45.89 71.68 134.01<br>162.45 246.65 281.45<br>345.85 392.40 499.92<br>532.09 619.89 663.29<br>725.86 827.09 854.15<br>950.34 972.28 997.72<br>1018.72 1089.34 1152.63<br>1187.38 1206.16 1235.62<br>1245.41 1270.49 1303.35

		H -1.076087 -2.168102 0.789003<br>O 2.296436 0.408256 -0.283162<br>C -2.474228 0.270211 -0.161993<br>H -3.008244 0.163813 0.794500<br>H -2.520103 .322302 -0.449616<br>H -3.018319 -0.322628 -0.901816<br>O 0.035084 2.002428 -0.056634<br>H 0.952680 2.215331 -0.272097			1339.33 1361.67 1407.07<br>1429.08 1454.14 1479.74<br>1486.53 1492.86 1880.93<br>2883.13 2995.29 3009.68<br>3066.51 3087.78 3088.38<br>3139.75 3149.42 3813.59
$C_6H_9O_2$	INT 2C	C -1.065126 -0.175037 -0.048142<br>C 0.030029 0.699098 0.458769<br>C 1.264289 0.103938 0.055739<br>C 0.914389 -1.579488 0.061075<br>C -0.627577 -1.613555 -0.046939<br>H 0.016786 0.741120 1.568808<br>H 1.430484 -2.080405 -0.757594<br>H 1.278108 -2.018186 0.994283<br>H -0.958114 -2.122456 -0.958159<br>H -1.076087 -2.168102 0.789003<br>O 2.296436 0.408256 -0.283162<br>C -2.474228 0.270211 -0.161993<br>H -3.008244 0.163813 0.794500<br>H -2.520103 1.322302 -0.449616<br>H -3.018319 -0.322628 -0.901816<br>O 0.035084 2.002428 0.056634<br>H 0.952680 2.215331 -0.272097	-90.11	0.098<br>0.068<br>0.042	45.89 71.68 134.01<br>162.45 246.65 281.45<br>345.85 392.40 499.92<br>532.09 619.89 663.29<br>725.86 827.09 854.15<br>950.34 972.28 997.72<br>1018.72 1089.34 1152.63<br>1187.38 1206.16 1235.62<br>1245.41 1270.49 1303.35<br>1339.33 1361.67 1407.07<br>1429.08 1454.14 1479.74<br>1486.53 1492.86 1880.93<br>2883.13 2995.29 3009.68<br>3066.51 3087.78 3088.38<br>3139.75 3149.42 3813.59
$C_6H_9O_2$	INT 3C	C 1.441845 -0.034463 -0.010191<br>C 0.444258 -0.939169 -0.043705<br>C -1.004559 -0.765665 0.137738<br>C -1.614987 0.564025 0.574039<br>C -1.931167 1.409254 -0.614985<br>H 0.707299 -1.977269 -0.233182<br>H -2.536678 0.293848 1.105351<br>H -0.971247 1.092751 1.279085<br>H -2.336012 0.943356 -1.503468<br>H -1.976436 2.486552 -0.532223<br>O -1.742430 -1.707982 -0.065850<br>C 1.377368 1.451094 0.166806<br>H 0.482993 1.875351 -0.282407<br>H 1.403897 1.706761 1.229386<br>H 2.257032 1.889142 -0.303915<br>O 2.731404 -0.422014 -0.156994<br>H 2.780814 -1.380979 -0.234090	-31.02	0.105<br>0.047<br>0.035	34.68 125.74 140.18<br>162.84 188.93 236.65<br>271.02 341.56 423.92<br>481.71 508.14 542.11<br>564.87 581.37 712.52<br>783.35 859.23 876.11<br>933.37 1034.28 1040.59<br>1062.88 1093.85 1170.93<br>1228.31 1270.19 1292.44<br>1359.52 1397.94 1450.90<br>1459.28 1471.01 1485.26<br>1508.66 1715.39 1783.87<br>3029.63 3076.29 3123.89<br>3150.24 3168.85 3172.58<br>3189.94 3282.39 3868.88
$C_6H_9O_2$	INT 4C	C -0.806153 0.501844 -0.124181<br>C -1.524236 -0.495589 -0.634092<br>C 1.956574 -0.726035 -0.201089<br>C 1.696525 0.771960 -0.250565<br>C 0.345676 1.072378 -0.908366<br>H -1.313318 -0.905951 -1.617540<br>H 1.765442 1.172561 0.766044<br>H 2.517395 1.192932 -0.839691<br>H 0.237047 2.158912 -0.991561<br>H 0.350847 0.666186 -1.923184<br>O 2.476439 -1.349752 0.645666<br>C -1.079148 1.064928 1.242161<br>H -1.103886 2.158356 1.208610<br>H -2.030768 0.705013 1.630566	-43.04	0.099<br>0.040<br>0.036	23.48 43.13 108.13<br>130.25 192.24 220.47<br>279.48 296.22 384.00<br>402.33 469.27 537.43<br>594.47 787.63 814.30<br>859.99 905.56 970.09<br>1032.86 1039.57 1060.19<br>1145.48 1193.82 1213.99<br>1232.69 1306.83 1327.59<br>1377.55 1395.96 1433.30<br>1450.52 1480.56 1494.25<br>1500.27 1795.54 1993.85<br>3048.26 3054.58 3059.49

		H -0.297472 0.777628 1.953095<br>O -2.566187 -1.061144 0.046769<br>H -2.942737 -1.775390 -0.469028			3100.59 3105.79 3115.73<br>3162.61 3181.01 3942.08
$C_6H_9O_2$	INT 5C	C 1.090133 0.128007 -0.004526<br>C 0.021730 -0.657970 0.097762<br>C -1.257098 0.125195 0.284951<br>C -0.855442 1.561935 -0.091617<br>C 0.676928 1.582068 0.095772<br>H -1.558113 0.064516 1.357056<br>H -1.106606 1.703155 -1.144659<br>H -1.382333 2.315890 0.491547<br>H 1.173716 2.196915 -0.659888<br>H 0.951846 1.995842 1.072935<br>O -2.347671 -0.413205 -0.332182<br>C 2.511502 -0.303439 -0.150323<br>H 3.108069 0.021449 0.707865<br>H 2.581611 -1.388681 -0.227397<br>H 2.961321 0.140892 -1.043162<br>O -0.019175 -2.010359 0.103223<br>H -0.921263 -2.276243 -0.114733	-21.53	0.100<br>0.066<br>0.042	108.04 153.44 166.69<br>247.27 251.76 302.13<br>409.27 443.39 480.73<br>546.83 593.50 662.80<br>729.39 838.95 924.44<br>963.26 986.22 1023.75<br>1040.01 1064.83 1107.66<br>1133.35 1184.20 1226.54<br>1231.65 1259.21 1281.05<br>1319.79 1330.79 1380.33<br>1422.69 1487.70 1489.01<br>1493.17 1508.07 1819.45<br>2860.12 3044.18 3049.51<br>3086.57 3099.53 3102.14<br>3152.01 3154.98 3832.78
$C_6H_9O_2$	INT 6C	C -1.094997 0.117618 0.317998<br>C -0.016367 -0.823145 -0.235645<br>C 1.345220 0.065658 -0.079522<br>C 0.892358 1.488276 0.213374<br>C -0.604975 1.513614 -0.084105<br>H -0.060355 -0.866467 -1.348113<br>H 1.084087 1.648570 1.280089<br>H 1.508366 2.189855 -0.349259<br>H -1.127124 2.306742 0.451717<br>H -0.776572 1.669742 -1.154534<br>O 2.442282 -0.352637 -0.201257<br>C -2.487977 -0.229026 -0.184266<br>H -2.522919 -0.184269 -1.277095<br>H -2.770682 -1.237170 0.124194<br>H -3.231001 0.470428 0.205916<br>O 0.152170 -1.999794 0.322514<br>H -1.058987 0.024050 1.410021	-51.00	0.105<br>0.064<br>0.042	60.37 180.10 231.56<br>233.49 279.13 319.15<br>448.57 464.95 510.71<br>533.72 595.30 642.90<br>780.05 843.46 913.01<br>949.88 961.09 1010.05<br>1051.97 1098.65 1116.45<br>1142.92 1176.40 1231.07<br>1254.68 1292.62 1305.26<br>1315.16 1347.53 1397.57<br>1413.79 1449.42 1497.84<br>1502.75 1505.10 1931.68<br>2871.47 3052.13 3055.00<br>3065.57 3071.68 3124.43<br>3134.09 3141.88 3147.79
$C_6H_9O_2$	INT 7C	C 1.138095 0.143109 0.394441<br>C -0.084430 -0.660502 0.134180<br>C -1.261510 0.121403 -0.050002<br>C -0.862757 1.581349 0.081388<br>C 0.673485 1.552870 -0.058021<br>H 1.325506 0.147512 1.478630<br>H -1.360766 2.199165 -0.665985<br>H -1.174841 1.938891 1.068027<br>H 0.943368 1.677565 -1.110426<br>H 1.167837 2.342866 0.507419<br>O -2.375727 -0.367496 -0.240861<br>C 2.388228 -0.367390 -0.317814<br>H 3.232710 0.297399 -0.124765<br>H 2.651588 -1.369808 0.023117<br>H 2.219707 -0.406153 -1.396877<br>O -0.113605 -1.984044 0.134778<br>H -1.037116 -2.240151 -0.035509	-163.25	0.101<br>0.067<br>0.043	103.10 159.51 210.18<br>226.47 282.43 313.19<br>441.43 515.29 565.67<br>579.52 647.00 671.95<br>727.47 822.12 909.79<br>959.06 987.31 1018.32<br>1070.19 1099.58 1122.01<br>1189.81 1216.93 1245.87<br>1300.40 1307.78 1317.77<br>1347.13 1409.04 1469.61<br>1476.25 1497.22 1505.25<br>1507.08 1550.59 1682.30<br>3000.40 3062.14 3069.48<br>3077.04 3134.70 3136.71<br>3142.75 3148.55 3719.65
$C_6H_9O_2$	TS1C	C -0.891819 0.004528 -0.382656<br>C 0.141124 -0.871840 -0.555272	-6.70	0.110<br>0.051	-362.95, 95.08 129.42<br>179.21 187.78 211.01

		C 1.424367 -0.223522 -0.212987<br>C 1.104944 1.191240 0.264321<br>C -0.381931 1.389466 -0.052671<br>H 0.063544 -1.901608 -0.881269<br>H 1.286462 1.220022 1.340367<br>H 1.771076 1.907937 -0.215568<br>H -0.951416 1.813983 0.775014<br>H -0.531968 2.031558 -0.926912<br>O 2.520809 -0.719363 -0.293294<br>C -2.319581 -0.214231 -0.749317<br>H -2.526471 0.283994 -1.701661<br>H -2.551841 -1.274009 -0.853280<br>H -2.969572 0.222198 0.009862<br>O -0.922901 -0.581928 1.656617<br>H -0.835698 -1.547590 1.678348		0.049	253.70 303.09 390.89<br>488.44 535.59 575.82<br>646.15 715.06 807.07<br>839.36 854.96 888.42<br>984.72 1001.53 1028.56<br>1044.37 1166.42 1172.83<br>1190.52 1244.86 1285.47<br>1311.32 1363.77 1410.99<br>1448.99 1474.09 1480.91<br>1486.91 1594.54 1834.21<br>3063.45 3069.90 3102.90<br>3131.87 3135.63 3156.28<br>3165.60 3231.41 3813.07
$C_6H_9O_2$	TS2C	C -1.073337 0.134591 -0.297026<br>C -0.007703 -0.649262 -0.635084<br>C 1.261615 0.078438 -0.356230<br>C 0.897107 1.331629 0.433769<br>C -0.637041 1.358227 0.461910<br>H -0.051959 -1.533819 -1.256402<br>H 1.334426 1.241476 1.428628<br>H 1.339214 2.202988 -0.051007<br>H -1.035058 1.298475 1.480630<br>H -1.060177 2.260263 0.009431<br>O 2.366904 -0.260337 -0.689418<br>C -2.506731 -0.204119 -0.470904<br>H -2.648982 -1.034199 -1.161692<br>H -2.914464 -0.495649 0.504046<br>H -3.078088 0.661673 -0.815329<br>O 0.091805 -1.584103 1.201714<br>H 0.841946 -2.142713 0.944721	2.43	0.101<br>0.056<br>0.047	-442.95, 73.08 85.91<br>100.54 125.89 161.90<br>213.82 295.65 430.88<br>488.28 538.51 575.36<br>642.34 757.47 804.78<br>852.89 886.20 903.73<br>979.68 1002.74 1032.79<br>1046.27 1169.13 1171.28<br>1190.02 1242.76 1282.52<br>1308.77 1362.74 1408.34<br>1445.68 1471.23 1473.62<br>1480.25 1608.52 1856.44<br>3048.12 3060.05 3093.96<br>3106.37 3110.80 3155.11<br>3162.85 3241.96 3808.33
$C_6H_9O_2$	TS3C	C 1.074996 -0.312337 0.037860<br>C -0.095661 -1.042310 -0.103515<br>C -1.387247 -0.393963 0.089245<br>C -1.300659 1.138365 -0.094322<br>C -0.035305 1.467405 -0.822707<br>H -0.084622 -2.020170 -0.574227<br>H -2.202178 1.429765 -0.641657<br>H -1.356261 1.625051 0.882979<br>H 0.000757 1.248908 -1.884477<br>H 0.582514 2.296098 -0.498623<br>O -2.439225 -0.968176 0.257858<br>C 1.457750 0.491351 1.255014<br>H 0.588608 0.909085 1.757427<br>H 1.951052 -0.198682 1.946188<br>H 2.163182 1.280356 0.996952<br>O 2.202445 -0.704755 -0.608854<br>H 1.967939 -1.278029 -1.346045	33.17	0.111<br>0.052<br>0.045	-609.75, 106.84 192.63<br>235.82 294.87 312.88<br>382.19 432.87 449.22<br>490.41 517.96 563.13<br>583.47 611.96 699.53<br>778.60 808.53 846.18<br>917.91 1027.29 1030.04<br>1044.30 1079.56 1182.34<br>1199.99 1220.34 1269.15<br>1281.74 1379.62 1426.10<br>1458.91 1474.72 1481.90<br>1489.39 1555.33 1796.48<br>3067.18 3070.17 3113.13<br>3144.33 3151.32 3195.76<br>3196.34 3251.82 3874.21
$C_6H_9O_2$	TS4C	C 0.599584 -0.865754 -0.238041<br>C 0.840685 0.368141 -0.792053<br>C -1.040683 0.879092 -0.022405<br>C -1.750830 -0.475237 -0.019167<br>C -0.726806 -1.442478 -0.662648<br>H 0.563607 0.570812 -1.823728<br>H -1.963339 -0.753321 1.018403	19.69	0.080<br>0.072<br>0.049	-558.78, 67.08 108.22<br>138.97 168.83 205.28<br>269.07 356.61 360.64<br>450.37 519.28 594.33<br>606.39 789.87 793.28<br>875.52 916.36 957.85<br>989.35 1020.09 1031.48

		H -2.703937 -0.389477 -0.546595<br>H -0.866663 -2.465448 -0.312643<br>H -0.837328 -1.439692 -1.749679<br>O -1.138332 1.780946 0.736345<br>C 1.204150 -1.300448 1.057513<br>H 1.479893 -2.358082 1.014103<br>H 2.090064 -0.714099 1.296193<br>H 0.488141 -1.190637 1.884237<br>O 1.809209 1.177775 -0.263525<br>H 1.625950 2.090278 -0.502038			1128.07 1184.01 1199.16<br>1221.59 1274.52 1285.42<br>1324.09 1378.83 1419.14<br>1463.84 1480.98 1487.36<br>1501.53 1614.27 1903.96<br>3014.96 3059.12 3082.38<br>3087.53 3117.12 3142.59<br>3151.32 3166.23 3904.61
$C_6H_9O_2$	TS5C	C 1.114402 0.114681 -0.024082<br>C -0.026953 -0.665786 0.049043<br>C -1.267132 0.146947 0.071614<br>C -0.815207 1.592426 -0.135568<br>C 0.710805 1.564609 0.106807<br>H -0.709086 -0.108896 1.274300<br>H -1.041375 1.835442 -1.177135<br>H -1.354468 2.291632 0.500899<br>H 1.260376 2.193937 -0.597296<br>H 0.956244 1.929859 1.112808<br>O -2.394181 -0.354878 -0.079226<br>C 2.515899 -0.370030 -0.084015<br>H 3.049171 -0.132350 0.844672<br>H 2.551391 -1.449429 -0.232409<br>H 3.062256 0.116779 -0.897335<br>O -0.119064 -1.993077 0.006977<br>H -1.059421 -2.190418 -0.153303	32.50	0.103<br>0.066<br>0.042	-990.53, 113.64 135.55<br>178.77 235.01 267.35<br>302.51 460.50 515.61<br>552.41 601.70 634.64<br>714.37 730.87 829.04<br>920.81 986.60 991.32<br>1014.39 1042.11 1084.01<br>1112.00 1171.31 1223.80<br>1242.56 1276.53 1306.19<br>1347.62 1364.09 1410.20<br>1435.14 1474.69 1479.64<br>1484.21 1491.41 1587.10<br>1680.93 3028.61 3032.71<br>3086.75 3090.05 3100.85<br>3153.12 3159.22 3699.90
$C_6H_9O_2$	TS6C	C -1.074577 0.099453 0.018754<br>C 0.021915 -0.917397 -0.189412<br>C 1.317292 -0.111085 -0.239182<br>C 1.050418 1.203354 0.484477<br>C -0.432637 1.474184 0.188731<br>H -0.071181 -1.682272 -0.959988<br>H 1.210156 1.035654 1.553349<br>H 1.732843 1.980483 0.146112<br>H -0.930524 2.077664 0.948235<br>H -0.529023 2.008150 -0.765364<br>O 2.326931 -0.433741 -0.798754<br>C -2.409764 -0.055910 -0.652411<br>H -2.323989 0.202567 -1.714096<br>H -2.766266 -1.085076 -0.583450<br>H -3.154838 0.602587 -0.202513<br>O -0.191211 -1.415770 1.137152<br>H -1.088820 -0.499257 1.204785	53.78	0.108<br>0.059<br>0.049	-1840.63, 93.48 157.92<br>223.20 241.83 273.06<br>341.05 465.02 524.68<br>550.01 623.32 692.74<br>791.28 857.83 875.86<br>943.73 961.95 1002.52<br>1027.38 1060.83 1091.86<br>1140.64 1170.10 1197.35<br>1218.55 1260.67 1284.62<br>1297.02 1307.61 1368.49<br>1410.69 1461.75 1488.92<br>1489.93 1496.79 1892.39<br>1955.91 3038.16 3043.83<br>3088.16 3117.95 3127.32<br>3134.10 3148.42 3167.45
$C_6H_9O_2$	TS7C	C 1.121353 0.215607 0.148506<br>C -0.056457 -0.676040 0.155805<br>C -1.290182 0.077625 -0.001255<br>C -0.923773 1.554765 0.121342<br>C 0.595796 1.598490 -0.159995<br>H 0.348467 -0.261596 1.256804<br>H -1.513779 2.152363 -0.571790<br>H -1.159803 1.893883 1.135505<br>H 0.761563 1.780214 -1.232998<br>H 1.112147 2.388015 0.387758<br>O -2.389193 -0.425998 -0.158624<br>C 2.512015 -0.256968 -0.078210	44.91	0.101<br>0.066<br>0.041	-1886.87, 119.61 150.85<br>169.32 215.16 256.18<br>271.80 364.25 456.32<br>510.41 524.77 600.16<br>660.61 694.27 808.03<br>912.16 971.44 987.86<br>1023.22 1042.87 1107.00<br>1143.89 1203.29 1226.23<br>1244.64 1267.77 1300.10<br>1341.98 1406.62 1447.46<br>1467.69 1477.11 1482.23<br>1487.98 1513.57 1768.53

		H 3.242995 0.421501 0.366331<br>H 2.659086 -1.255488 0.336957<br>H 2.725212 -0.320790 -1.155246<br>O 0.010814 -2.016298 -0.043035<br>H -0.901370 -2.340602 -0.027213			2175.56 2993.80 3002.69<br>3066.97 3095.91 3116.71<br>3144.65 3152.66 3795.30
$C_6H_9O_2$	TS8C	C 0.835389 -0.073099 -0.412528<br>C -0.218044 -0.959997 -0.456222<br>C -1.444645 -0.270539 -0.084311<br>C -1.094120 1.200282 0.179398<br>C 0.355077 1.356948 -0.296906<br>H -0.163031 -2.020229 -0.664736<br>H -1.802057 1.849363 -0.335334<br>H -1.197430 1.386194 1.251495<br>H 0.404821 1.797802 1.296673<br>H 0.984003 1.955980 0.360922<br>O -2.555845 -0.743740 0.013031<br>C 1.407944 -0.370072 1.689667<br>H 0.553052 -0.056419 2.276793<br>H 1.634615 -1.428904 1.714979<br>H 2.256617 0.301753 1.653626<br>O 2.066738 -0.245900 -0.948935<br>H 2.192649 -1.169554 -1.188430	7.38	0.110<br>0.050<br>0.048	-680.79, 99.71 135.74<br>164.32 233.84 254.70<br>349.41 384.27 442.15<br>504.64 535.34 549.48<br>580.90 605.66 643.57<br>780.62 809.67 855.37<br>908.53 949.82 1012.66<br>1021.31 1150.04 1155.16<br>1206.78 1221.99 1250.32<br>1283.81 1326.44 1420.73<br>1427.91 1430.76 1466.92<br>1497.88 1549.79 1798.90<br>3082.00 3092.62 3104.25<br>3140.62 3151.99 3239.84<br>3267.77 3273.10 3880.45
$C_6H_9O_2$	TS9C	C 1.071069 0.144632 -0.049838<br>C -0.009467 -0.668416 0.096324<br>C -1.267625 0.108525 -0.029373<br>C -0.893576 1.577251 -0.008512<br>C 0.648008 1.589445 0.033878<br>H -0.236737 -0.389820 1.892026<br>H -1.296971 2.057051 -0.901601<br>H -1.354414 2.053344 0.858392<br>H 1.080059 2.167355 -0.787581<br>H 1.022695 2.032830 0.963950<br>O -2.353386 -0.400335 -0.155834<br>C 2.490868 -0.285403 -0.114320<br>H 2.999342 0.049054 0.828092<br>H 2.568108 -1.358820 -0.286279<br>H 3.023067 0.245032 -0.908510<br>O -0.031408 -2.009406 0.002963<br>H -0.962459 -2.276198 -0.004484	18.65	0.101<br>0.067<br>0.042	- 891.46, 106.31 115.76<br>131.29 241.83 278.21<br>292.53 404.19 451.75<br>485.17 517.35 540.72<br>577.97 651.97 669.50<br>732.83 820.78 928.87<br>973.59 1005.31 1041.33<br>1056.70 1143.01 1172.33<br>1236.26 1239.32 1266.20<br>1316.57 1350.76 1398.93<br>1441.69 1453.21 1481.54<br>1484.99 1487.66 1692.47<br>1849.62 3040.10 3047.94<br>3092.77 3101.25 3101.38<br>3146.70 3158.21 3791.15
$C_6H_9O_2$	TS10C	C -1.071857 0.266345 -0.105248<br>C 0.010840 -0.718711 -0.458040<br>C 1.280531 0.071162 -0.126485<br>C 0.938867 1.551620 -0.144376<br>C -0.555342 1.498349 0.096052<br>H 0.000365 -0.905939 -1.546340<br>H 1.509504 2.094551 0.609730<br>H 1.182436 1.972301 -1.126725<br>H -0.435425 1.539329 2.034440<br>H -1.158151 2.389475 0.223858<br>O 2.313140 -0.448142 0.192123<br>C -2.489716 -0.163399 0.025601<br>H -2.854419 -0.591607 -0.912905<br>H -2.565081 -0.947202 0.784438<br>H -3.131922 0.671059 0.308541<br>O -0.070742 -1.931410 0.242477<br>H 0.833587 -2.217751 0.423130	61.91	0.102<br>0.067<br>0.043	-727.30, 71.69 153.63<br>201.94 211.38 244.72<br>277.80 319.33 390.50<br>426.28 473.14 541.87<br>571.71 661.75 703.39<br>806.49 866.18 904.89<br>972.26 987.97 1031.32<br>1057.76 1090.45 1156.78<br>1203.99 1220.80 1230.47<br>1293.50 1319.09 1353.65<br>1403.25 1423.98 1460.64<br>1474.75 1489.07 1667.50<br>1893.93 2964.89 3055.06<br>3060.57 3114.21 3134.38<br>3153.04 3216.05 3828.14

$C_5H_6O_2$	P1C	C 1.054962 -0.186619 0.000042<br>C 0.017872 -1.041439 0.000089<br>C -1.232856 -0.273139 -0.000007<br>C -0.869210 1.217607 0.000327<br>C 0.664208 1.262239 -0.000344<br>H 0.055089 -2.122097 0.000207<br>H -1.311977 1.686947 -0.879033<br>H -1.310990 1.686055 0.880679<br>H 1.084201 1.755375 -0.880199<br>H 1.085019 1.756132 0.878682<br>O -2.358654 -0.706491 -0.000194<br>O 2.367460 -0.447905 0.000126<br>H 2.518366 -1.399140 -0.000437	-42.48	0.201<br>0.070<br>0.053	97.67 171.12 360.40<br>461.28 509.77 535.73<br>567.34 590.36 636.37<br>824.26 855.65 855.83<br>919.12 1018.39 1023.63<br>1158.14 1169.51 1225.09<br>1238.59 1246.01 1284.97<br>1331.40 1450.68 1460.98<br>1491.85 1698.27 1844.74<br>3083.38 3103.69 3120.79<br>3150.88 3247.45 3873.84
$C_6H_8O_2$	P2C	C 1.062464 0.125891 -0.000031<br>C -0.012767 -0.672663 0.000149<br>C -1.260966 0.105767 -0.000016<br>C -0.886396 1.574533 0.000530<br>C 0.653998 1.581716 -0.000355<br>H -1.318740 2.056309 -0.878082<br>H -1.317315 2.054828 0.880679<br>H 1.061133 2.091754 -0.878536<br>H 1.062321 2.092746 0.876684<br>O -2.362535 -0.394632 -0.000459<br>C 2.489092 -0.302007 -0.000070<br>H 3.006741 0.090225 0.880529<br>H 2.572339 -1.388526 -0.001062<br>H 3.007215 0.091942 -0.879618<br>O -0.056696 -2.018104 0.000266<br>H -0.992397 -2.266817 -0.000286	-3.62	0.103<br>0.068<br>0.042	100.87 127.23 135.52<br>242.76 296.31 298.88<br>463.07 476.53 530.87<br>562.60 631.06 672.37<br>725.68 830.40 929.14<br>979.21 1008.15 1046.13<br>1064.32 1152.17 1170.77<br>1234.21 1236.67 1269.49<br>1320.14 1350.38 1401.16<br>1451.08 1459.42 1486.58<br>1486.67 1498.13 1782.46<br>1840.58 3052.76 3062.35<br>3092.79 3098.14 3107.38<br>3142.70 3158.78 3794.36
$C_6H_8O_2$	P3C	C 1.075215 0.310363 0.041323<br>C 0.007749 -0.675196 0.448499<br>C -1.273478 0.086040 0.099070<br>C -0.945220 1.569003 0.026133<br>C 0.554017 1.525539 -0.147942<br>H 0.035282 -0.823821 1.541579<br>H -1.488582 2.043952 -0.793246<br>H -1.253016 2.062232 0.955408<br>H 1.137034 2.402503 -0.403485<br>O -2.308916 -0.456053 -0.171000<br>C 2.499664 -0.116181 -0.069329<br>H 2.867089 -0.483744 0.893400<br>H 2.583998 -0.940954 -0.780783<br>H 3.134584 0.707150 0.397842<br>O 0.094394 -1.910866 -0.211726<br>H -0.807893 -2.229365 -0.339741	47.95	0.107<br>0.068<br>0.044	74.17 174.39 219.13<br>252.90 263.28 317.27<br>360.27 456.49 541.60<br>564.55 659.87 697.78<br>806.11 816.54 895.83<br>972.84 982.93 1035.68<br>1065.74 1094.82 1158.57<br>1205.44 1221.47 1230.39<br>1296.01 1324.92 1357.66<br>1407.39 1426.22 1460.68<br>1478.37 1494.91 1739.55<br>1891.55 2975.63 3057.38<br>3058.21 3119.29 3120.98<br>3153.46 3216.98 3830.27

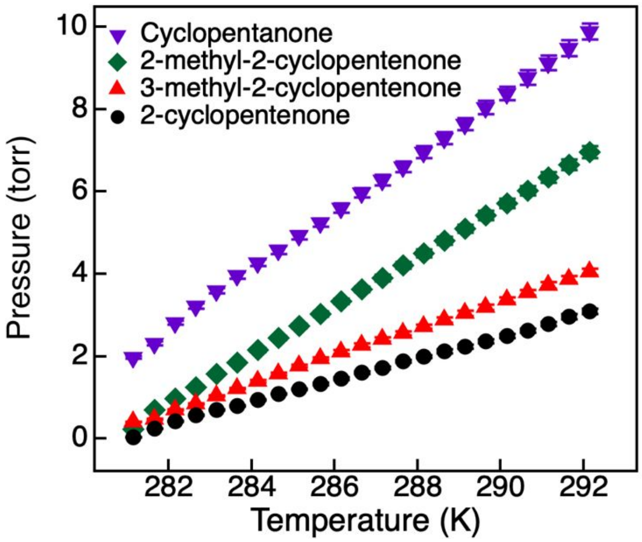

Figure S1. Measured vapor pressure for cylopentanone, 2-cyclopenten-1-one, 2-methyl-1-cyclopentenone, and 3-methyl-2-cyclopenten-1-one as a function of temperature. Each sample is placed in a vacuum sealed bubbler and freeze-pump-thawed to remove any solvent. The bubbler temperature is controlled using a regulated water bath. The pressure in the bubbler is measured using a 10-Torr pressure gauge (MKS) and the reported temperature are those of the circulated water bath. The error bars are 2-standard deviation uncertainty from the average of 3 independent datasets.

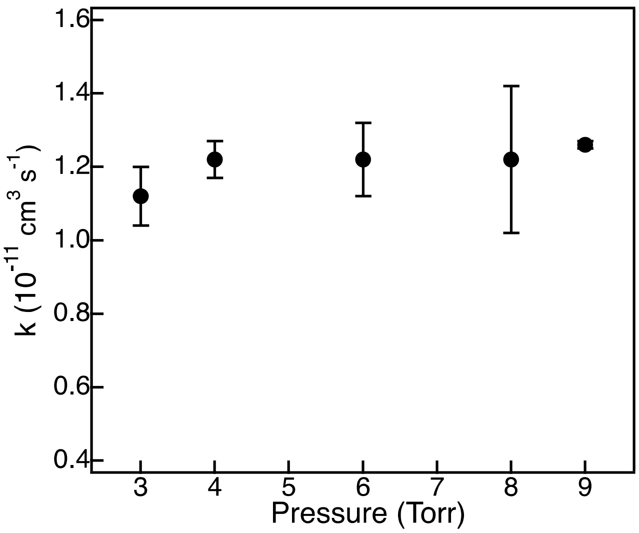

Figure S2. Pressure dependence of the OH radical with 2-cyclopenten-1-one at 300 K.

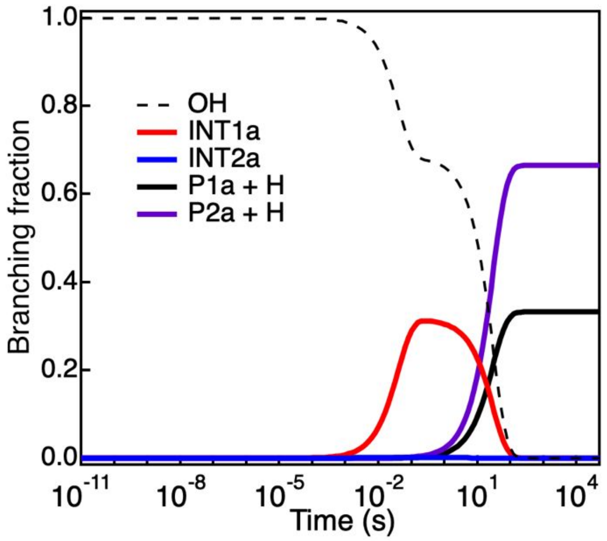

Figure S3. MESMER-calculated temporal profiles of INT1a (red line), INT2a (blue line), P1a (black line), and P2a (purple line) from reaction of the OH radical with cyclopentenone at 500 K and 5 Torr.

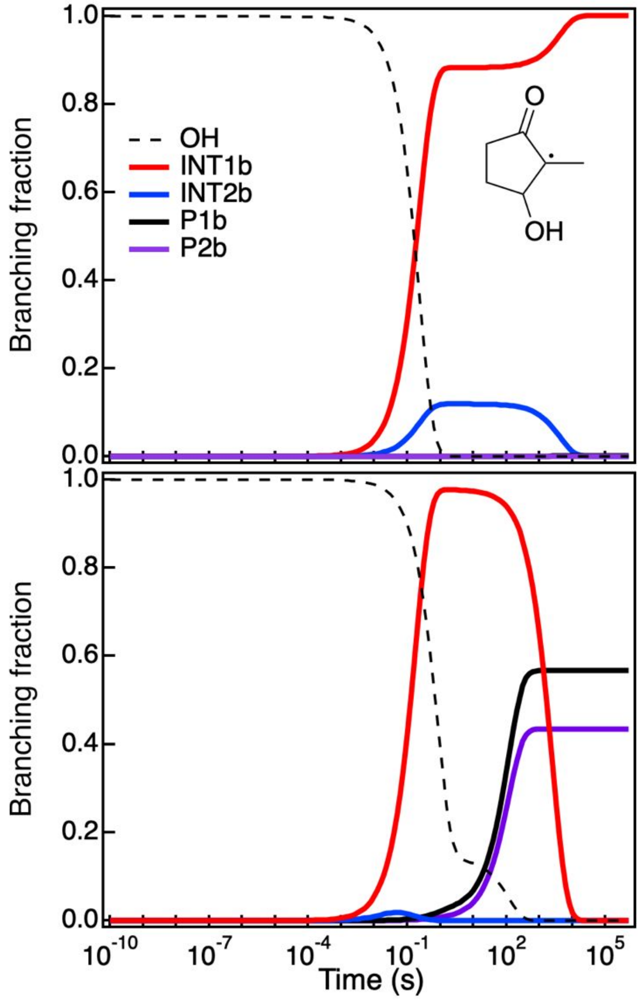

Figure S4. MESMER-calculated temporal profiles of INT1b (red line), INT2b (blue line), P1b (black line), and P2b (purple line) from reaction of the OH radical with 2-methyl-2-cyclopentenone at 293 K (top) and 500 K (bottom) and 5 Torr.

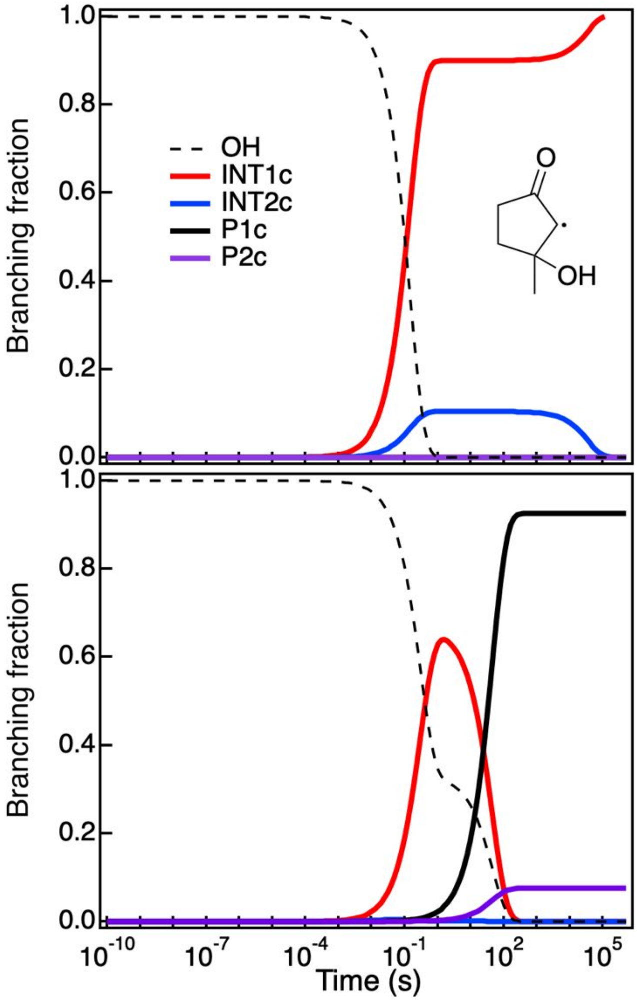

Figure S5. MESMER-calculated temporal profiles of INT1c (red line), INT2c (blue line), P1c (black line), and P2c (purple line) from reaction of the OH radical with 3-methyl-2-cyclopentenone at 293 K (top) and 500 K (bottom) and 5 Torr.
